# Θ-Net: A Deep Neural Network Architecture for the Resolution Enhancement of Phase-Modulated Optical Micrographs *In Silico*

**DOI:** 10.3390/s24196248

**Published:** 2024-09-26

**Authors:** Shiraz S. Kaderuppan, Anurag Sharma, Muhammad Ramadan Saifuddin, Wai Leong Eugene Wong, Wai Lok Woo

**Affiliations:** 1 Faculty of Science, Agriculture & Engineering (SAgE), Newcastle University, Newcastle upon Tyne NE1 7RU, UK; anurag.sharma@newcastle.ac.uk (A.S.); ramadan.saifuddin@newcastle.ac.uk (M.R.S.); 2 Engineering Cluster, Singapore Institute of Technology, 10 Dover Drive, Singapore 138683, Singapore; eugene.wong@singaporetech.edu.sg; 3 Computer and Information Sciences, Sutherland Building, Northumbria University, Northumberland Road, Newcastle upon Tyne NE1 8ST, UK; wailok.woo@northumbria.ac.uk

**Keywords:** deep neural networks, image denoising, computational phase-modulated nanoscopy, label-free optical imaging, biomedical imaging

## Abstract

Optical microscopy is widely regarded to be an indispensable tool in healthcare and manufacturing quality control processes, although its inability to resolve structures separated by a lateral distance under ~200 nm has culminated in the emergence of a new field named *fluorescence nanoscopy*, while this too is prone to several caveats (namely phototoxicity, interference caused by exogenous probes and cost). In this regard, we present a triplet string of concatenated O-Net (‘bead’) architectures (termed ‘Θ-Net’ in the present study) as a cost-efficient and non-invasive approach to enhancing the resolution of *non-fluorescent* phase-modulated optical microscopical images *in silico*. The quality of the afore-mentioned enhanced resolution (ER) images was compared with that obtained via other popular frameworks (such as ANNA-PALM, BSRGAN and 3D RCAN), with the Θ-Net-generated ER images depicting an increased level of detail (unlike previous DNNs). In addition, the use of cross-domain (transfer) learning to enhance the capabilities of models trained on differential interference contrast (DIC) datasets [where phasic variations are not as prominently manifested as amplitude/intensity differences in the individual pixels unlike phase-contrast microscopy (PCM)] has resulted in the Θ-Net-generated images closely approximating that of the expected (ground truth) images for both the DIC and PCM datasets. This thus demonstrates the viability of our current Θ-Net architecture in attaining highly resolved images under poor signal-to-noise ratios while eliminating the need for *a priori* PSF and OTF information, thereby potentially impacting several engineering fronts (particularly biomedical imaging and sensing, precision engineering and optical metrology).

## 1. Introduction

Over the past few decades, the field of artificial intelligence (AI) has witnessed considerable progress and deployments in numerous applications, such as computer vision [1,2], speech and text recognition [3,4] and cyber security [5,6], amongst others. Fueled primarily by developments in computing hardware resources (such as GPUs and memory [7]), a particular subset of AI algorithms (termed *deep neural networks* or DNNs for short) has played a fundamental role in driving this revolution. In this respect, it would be prudent to evaluate the impact of these DNNs in propelling developments in healthcare and ecological studies, two aspects which play prominent roles in circumventing present-day global dilemmas, such as pandemics [8] and climate change [9], amongst others. Of particular interest in this regard would be the role of these DNNs in image analysis [10], object detection and segmentation [11,12].

As a trusted primary approach formulated to address these healthcare and environmental issues, the role of optical microscopes cannot be undermined as they aid in the identification of host responses to disease-causing pathogens (e.g., the presence of tumors [13], impact of pathogens on native cellular metabolomics and molecular processes occurring *in vivo* [14,15], etc.), as well as the detection of ecologically essential microbiota (e.g., diatoms and phytoplankton, such as *Euglena* [16], coupled with soil microbes such as *P. alcaligenes* [17]) which play an essential role in the upkeep of marine and terrestrial ecosystems. Nonetheless, optical microscopes are plagued by a fundamental constraint—their lateral resolution (as they are often utilized) is confined to a minimum distance of 143nm, often described as the Abbe limit [18]. Numerous approaches (both optical and computational) have thus been proposed to circumvent this limitation. The former comprises mainly optical nanoscopic/super-resolution (SR) techniques [such as SIM [19], STED [20], PALM [21] and STORM [22] (amongst others)], realized through the addition of specialized but costly hardware attachments to the optical microscope, while the latter utilizes DNNs (such as ANNA-PALM [23], Deep-STORM [24], DeepZ [25] or 3D RCAN [26]) in seeking to enhance the resolution of micrographs acquired via conventional imaging techniques (often epi-fluorescence microscopy) *in silico*.

**Figure 1:**
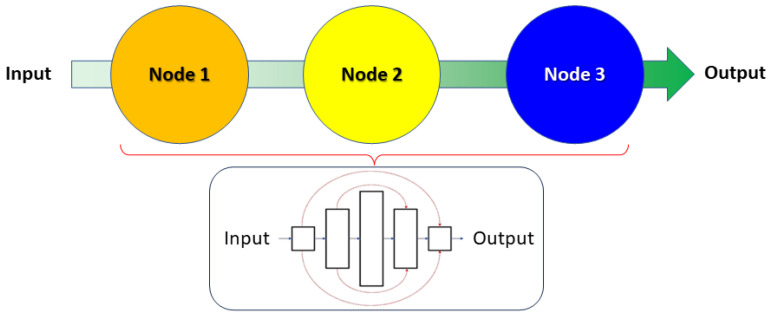
A generalized schematic (overview) of the Θ-Net architecture as proposed in the current context. Θ-Net adopts a ‘*string of beads*’ methodology of concatenating multiple O-Nets, thereby enhancing the DNN’s resilience to feature-based variations present in different samples that it might be trained with. Here (and as is presented in the current study), we employ a 3-node Θ-Net framework for model training and validation.

Despite the ubiquitous availability of DNN frameworks for SR imaging, we have come to realize that a number of the DNNs proposed (such as [23,24]) were developed to enhance the resolution of micrographs acquired via traditional widefield *epi-fluorescence microscopy*, where there is a clear distinction between the signal and the background (which is often dark). Such algorithms may thus also perform expectably well for other microscopical imaging modalities such as darkfield microscopy [27] or cross-polarized light microscopy [28], where the background is clearly demarcated by the extinction of optical signals. In contrast, for imaging modalities where there is little discrimination between the background and the signal being resolved, or where artifacts (such as halos or pseudo-relief features) are present in the image due to the imaging technique utilized (as is evident in DIC or PCM), some of these algorithms may *not* be suitable for increasing the resolution of the input images in this context (as depicted by the implementation of ANNA-PALM [23] in the Results Section of the present study). Consequentially, we have sought to develop a novel framework (named O-Net) which we described in our previously published study [29], although the potentiality of further improvements to the O-Net framework were noted to be plausible in its subsequent refinements. In this regard, we now propose an *extension* of the O-Net architecture (which we term Θ-Net) as a viable means of increasing the resolution of micrographs acquired through phase-modulated optical microscopical techniques (namely PCM and DIC microscopy). Θ-Net employs a concatenated architecture of multiple O-Nets (i.e., a computational ‘*string of beads*’), coupled with the transfer learning of features across these different phase-modulated microimaging modalities, so as to exploit the learning paradigms of phase-sensitive features resolvable through complementary phase microscopy techniques. Schematically, the Θ-Net architecture may be represented in Figure 1 as follows:

The testing of the proposed Θ-Net framework was performed using a separate dataset for each of the DIC and PCM imaging modalities. The results obtained in this respect (described in the following Figures 3–7 of the present study) seem promising, with ultrastructural details evident in these ER images.

## 2. Materials and Methods

### 2.1. Data Acquisition and Preparation (Light Microscopy)

The image datasets used for training were similar to those used for training O-Net, as expounded in our previous article in [29]. Generally, commercially available, prepared microscope slides (utilizing samples from a range of plant, animal and human tissues, as well as microbiota) were used for image acquisition to create the training datasets for the assessed DNNs. The images were acquired through 2 primary imaging modalities—PCM and DIC microscopy—using a Leica N PLAN L 20X/0.4 Corr Ph1 objective (Leica P/N: 506058) [for low-resolution (LR) images] and a Leica HCX PL Fluotar L 40X/0.60 Corr Ph2 objective (Leica P/N: 506203) [for high-resolution (HR) images] installed on a Leica DM4000M microscope, with a CMOS camera (RisingCam^®^ E3ISPM12000KPA, RisingCam, China) having a pixel size of 1.85 μm × 1.85 μm and an EK 14 Mot motorized stage (Märzhäuser Wetzlar GmbH & Co. KG, Wetzlar, Germany) mounted on it. The control of the motorized components of the microscope and camera settings was facilitated through a self-developed stage controller coupled with a desktop UI (also developed in C#). For each sample, similar regions of interest (ROIs) were imaged (under both PCM and DIC microscopes), with the acquired images registered and cropped using MATLAB R2020a (© 1984–2020, The MathWorks, Inc., Natick, MA, USA). Where present, shifts detected in the ROIs were mitigated through multi-layer image cropping using Corel PHOTO-PAINT X7 (© 2015 Corel Corporation), prior to splitting into 600 px × 600 px RGB image tiles in MATLAB R2020a (© 1984–2020, The MathWorks, Inc.). These tiles were then conjoined into LR-HR image pairs [the LR images being the **Source** (to be transformed) and the HR images being the **Expected** (*target/ground truth*) images], resulting in a total of 3944 image pairs for each imaging modality employed (i.e., DIC or PCM), before being downscaled to a 256 px × 512 px format and parsed into NumPy arrays for training the individual nodes of the proposed Θ-Net network in Python 3.8.

### 2.2. Θ-Net Architecture

The currently proposed framework (Θ-Net) utilizes a ‘*string of beads*’ architecture, comprising multiple (in this context, three) O-Nets as described in [29]. The O-Net models utilized for each of the nodes in the Θ-Net scaffold were selected after multiple empirical runs evaluating model architectures of varying depths and trained over a range of epochs (up to a maximum of 320 epochs). Further details pertaining to the individual model architectures are described in the following sub-sections.

#### 2.2.1. Generalized Θ-Net Structure

**Figure 2:**
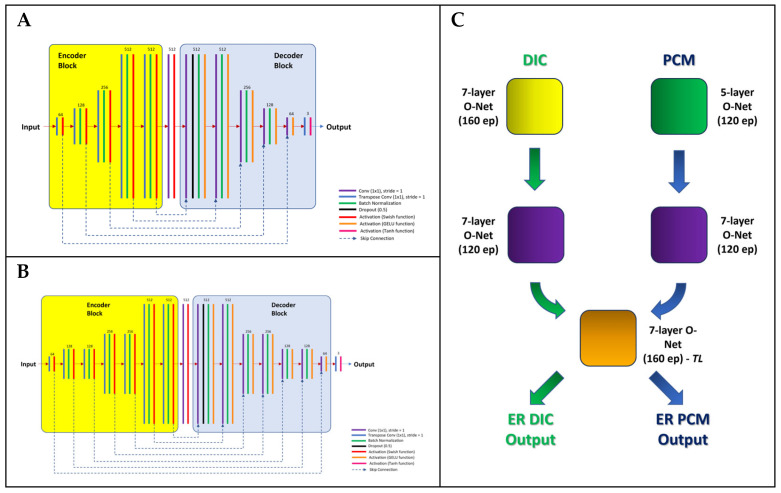
The structure of the 5-layer (Panel **A**) and 7-layer (Panel **B**) O-Net architectures utilized as nodes for Θ-Net (Panel **C**), as described in the present study. Each of the 3 nodes of Θ-Net (shown in Figure 1 previously) is an O-Net model specifically trained with the input image dataset for the imaging modality which it is intended to be deployed for use in. The exception here refers to the 3rd node utilized in the current Θ-Net framework—the O-Net model here was trained using both the DIC and PCM image datasets, via a *transfer learning* approach. As with the traditional O-Net architecture (described in [29]), skip-connections (*concatenations*) are used to join layers in the encoder block (consisting of transposed convolution operations) with their corresponding conjugates in the decoder block (comprising convolution operations).

In the present study, we attempted to enhance the resolution of the input DIC images by employing a 7-layer O-Net model trained with the DIC dataset for both the 1st and 2nd nodes and a 7-layer O-Net model trained on *both* the PCM and DIC datasets (the latter of which is *transfer-learnt*) for the 3rd node of the Θ-Net architecture. For the PCM images, we used a 5-layer O-Net model for the first node but a 7-layer O-Net model for the 2nd node. For the third node, we used the same transfer-learnt O-Net model as was used for the DIC images. Figure 2 below depicts a general schematic of the Θ-Net framework utilized in this regard.

At this juncture, it would be essential to highlight that the individual O-Net nodes within a Θ-Net chain are founded on the Pix2Pix GAN architecture, as discussed in [29,30]. Similarly, Swish [31] and GELU [32] activation functions were also incorporated into the encoder and decoder blocks, respectively (the mathematical definitions of these functions are included in the accompanying Supplementary Materials for the interested reader). An *adaptive moments estimation* (Adam) optimizer [33] was also employed for use in the GAN, the hyperparameters of which are as described in the associated Supplementary Materials.

The images generated by the Θ-Net models were also analyzed in MATLAB R2022b (© 1984–2022, The MathWorks, Inc.) using standard image quality metrics, such as the peak signal-to-noise ratio (PSNR), signal-to-noise ratio (SNR), image mean-squared error (IMSE) and structural similarity index (SSIM) (where plausible). The findings (and insights) gleaned from this assessment are henceforth depicted in the Results Section of this study.

#### 2.2.2. Cross-Domain Learning

An interesting aspect of the present study refers to the employment of *cross-domain learning* [34] across 2 commonly utilized phase-modulated optical microscopy techniques (i.e., PCM and DIC microscopy). Here, we demonstrate that it is possible to extricate information gleaned from images acquired under each of these techniques to *enhance* the overall performance of the Θ-Net models in computational nanoscopy (as exemplified in the Results Section of this study). This may potentially be attributable to **both** DIC and PCM translating *optical path length* (OPL) variations in the sample into amplitude (image brightness/intensity) differences, albeit via different routes (PCM translates differences in OPL *magnitude* into brightness variations, while DIC converts OPL *gradient* differences into 3D relief effects [35]). In this respect, we seek to utilize *transfer learning* as a vehicle to facilitate cross-domain learning between these 2 microscopical imaging modalities, thereby spelling benefits for the Θ-Net-generated ER images to incorporate the ‘best of both worlds’ while seeking to circumvent the limitations faced when employing each technique individually (e.g., birefringence artifacts encountered in DIC, as well as the lack of lateral and axial resolution coupled with unsuitability for specimens having high phase shifts for PCM imaging [35]).

#### 2.2.3. Comparative Networks for ER Image Analysis

Separately, we also attempted to include models derived from other industry-leading DNN frameworks (such as ANNA-PALM [23] and 3D RCAN [26], the latter of which is employed for *in silico* nanoscopy in the Aivia deep learning suite [36]), as a comparative performance gauge of the proposed Θ-Net models. Nonetheless, it would be apt to emphasize at this juncture (as was also highlighted in the Introduction Section earlier) that these frameworks were *specifically* adapted for increasing the resolution of *widefield epi-fluorescence monochrome/grayscale* microscopical images (3D RCAN [26] uses grayscale image Z-stacks for generating SR images); hence, a somewhat *close* (albeit not very similar) comparative assay in this respect would require splitting the current 2D RGB image into a 3-channel grayscale image stack, which would then be simulated as a Z-stack for training and validating the 3D RCAN [26]-based models. For ANNA-PALM [23] validation, we utilized the ImageJ A-Net plugin downloaded from [37] and the supplied models trained for increasing the resolution of microtubules within ImageJ 1.52n (NIH, USA), as the structures we wanted to enhance the resolution of generally resembled (in part) the filamentous strands characteristic of microtubules. In addition, models developed on a third comparative framework (termed BSRGAN [38]) were also included in the present study, for verification purposes.

### 2.3. Image Denoising

In addition to ER images, we also attempted to evaluate the capability of the proposed Θ-Net models for image denoising, despite the models not being trained specifically for this purpose. To conduct this, salt-and-pepper noise was synthetically introduced into the validation images using MATLAB R2022b (©1984–2022, The MathWorks, Inc.), and the assayed Θ-Net models were assessed on their propensity to denoise the noise-infused images.

The codes and models used to generate and evaluate the images presented in this study are available for download in the accompanying Supplementary Materials.

## 3. Results

The models developed using the presently assayed Θ-Net architecture were contrasted against our previous O-Net models [29], as well as other state-of-the-art *in silico* SR architectures, such as 3D RCAN [26], BSRGAN [38] and ANNA-PALM [23], for each of the DIC and PCM imaging modalities. The generated images obtained from each of these models are depicted in the subsequent figures.

### 3.1. DIC Imaging

**Figure 3:**
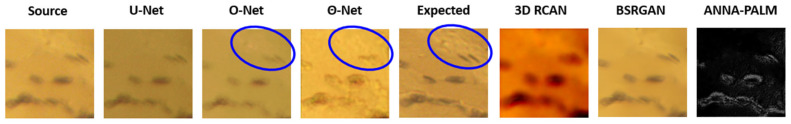
The validation of *in silico* ER images obtained through various models, including those developed using O-Net [29] and the presently surfaced Θ-Net. The sample shown here consists of highly magnified views of skeletal muscle tissue (L.S.) (adapted from Figure S11 of the Supplementary Materials, with further evaluation images being presented in this figure for the interested reader as well). The O-Net model was trained over 101 epochs, while the Θ-Net model assimilated O-Net models trained over 160 epochs (for the 1st node), a 120-epoch-trained O-Net model (for the 2nd node) and a 160-epoch-trained and transfer-learnt O-Net model (for the 3rd node). Notice the closer resemblance of the Θ-Net-generated image to the **Expected** (ground truth) image, as compared to the O-Net models (highlighted within the blue ellipses). *N.B.: The **Source** image (input) was acquired via a 20X/0.40 Ph1 objective, while the **Expected** (ground truth) image was obtained using a 40X/0.60 Ph2 objective. Images generated through models founded on other frameworks (namely 3D RCAN [26], BSRGAN [38] and ANNA-PALM [23]) were also included for comparison purposes (the ANNA-PALM [23] model developed for increasing the resolution of grayscale photomicrographs of microtubules was utilized as an extension within ImageJ 1.52n (NIH, USA), while the 3D RCAN [26] model was trained over 250 epochs with 1972 steps per epoch and 2 residual groups). *N.B.: The Θ-Net models utilized for generating the ER images in this figure and Figure S11 implement optional *node scaling* for each node, differing from the rest of this study. The supplied code (described in the accompanying Supplementary Materials) allows the user to select whether node scaling should be applied (or not), based on the user’s discernment of their image dataset and acquisition parameters.

From Figure 3 and its associated Figure S11 in the accompanying Supplementary Materials, it may be clearly discerned that images generated via the Θ-Net-trained models exhibit an enhanced contrast and, consequently, an increased resolution of details and features as compared to those generated from the O-Net-trained models. Nonetheless, pseudo-relief depictions of ER features are also apparent in the Θ-Net-generated images, although network hallucination (a prominent flaw in DNN-generated images as highlighted in [39]) is not clearly evident in both the O-Net- and the Θ-Net-generated images. In some respects, it may be argued that the Θ-Net ER images provide greater clarity than even the **Expected** images, suggesting an *enhanced* feature detection realized through the application of the Θ-Net models, putatively due to the effect of the transfer learning of features from the PCM dataset.

**Figure 4:**
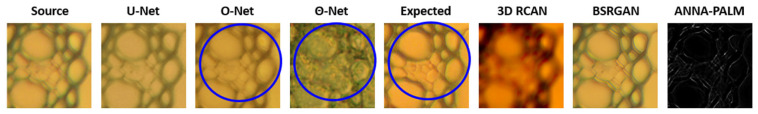
*In silico* ER images obtained through several models, including those developed using U-Net [11], O-Net [29] and Θ-Net (similar O-Net and Θ-Net models as described in Figure 3 previously were used for the ER images here as well). As with Figure 3, the **Source** image (input) was acquired via a 20X/0.40 Ph1 objective, while the **Expected** (ground truth) image was obtained using a 40X/0.60 Ph2 objective. *N.B.: This figure was sourced from Figure S12 of the accompanying Supplementary Materials. Notice that some features (such as the cell walls within the blue ellipses as shown here or the shadow-like striations within the green ellipses in Figure S12) are resolved differently in the Θ-Net models as compared to that from O-Net—in the Θ-Net images, the cell walls appear more granular around the periphery, while the striations are less visible. This might be postulated to be due to the Θ-Net architecture adopting a transfer-learnt model from PCM; hence, pseudo-relief artifacts characteristic of DIC imaging would be less pronounced (leading to a reduced accentuation of the striations), while phasic variations identified in the image are characterized pixel-wise (accounting for the granular edges of the cell wall when adopting Θ-Net models for ER images). *N.B.: The deployment of the Θ-Net model utilized for generating the ER images in this figure does *not* employ node scaling, differing from that used for Figure 3.

Figure 4 below showcases the validation of both the O-Net and Θ-Net models with an *untrained* image dataset (comprising images which the models were never trained with). In addition, models adopting the popular U-Net architecture, 3D RCAN [26], BSRGAN [38] and ANNA-PALM [23] were also included (the said models representing the industry standard for most *in silico* ER image applications). Notably, it may be observed that although both the O-Net and Θ-Net models seemingly outperform models adopting the U-Net [11], 3D RCAN [26], BSRGAN [38] and ANNA-PALM [23] architectures (in terms of the details resolvable visually), *none* of the models *may* be completely absolved from network hallucination artifacts, although this is subject to further evaluation and discussion (as detailed in a later section of this study).

**Figure 5:**
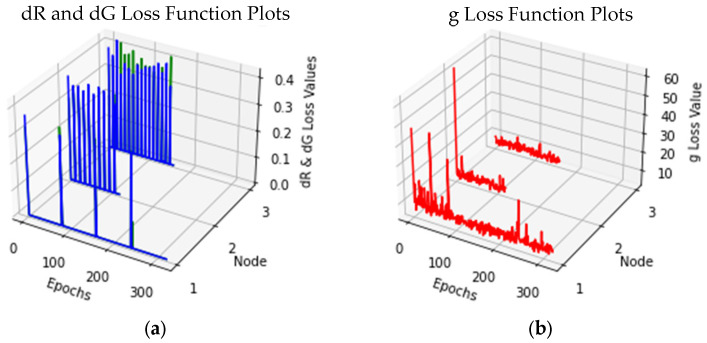
Loss functions for DIC micrographs imaged under the Θ-Net architecture. (**a**) Discriminator losses on *both* the real and generated samples, represented by dR (green line) and dG (blue line) losses, respectively, as well as (**b**) the generator (g) loss plotted as a red line. The spikes observed in the dR and dG loss function plots (**a**) demonstrate the commencement of a subsequent training run, implicating the selection of a new random seed for DNN training. Moreover, this approach of intermittently training the DNN models over multiple runs (rather than a single continuous run) is preferred [40] as it *restructures* the loss function error landscape, allowing the model to reach its global minimum (even if it may be trapped in one of the local minima during an earlier training run).

Approaching from a computational standpoint, we show the plots of the respective loss functions, namely the discriminator losses on real samples (dR) and generated samples (dG) and the generator (g) loss for the training runs in the various nodes of the Θ-Net models in Figure 5.

Conversely, an analysis of the images acquired via PCM revealed the following observations (detailed in the subsequent sections).

### 3.2. PCM Imaging

**Figure 6:**
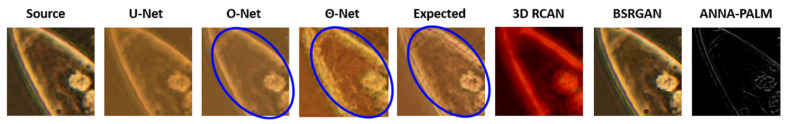
A comparison of the images obtained through utilizing models developed on the O-Net and Θ-Net architectures using PCM micrographs. Here, the O-Net model was trained over 120 epochs, while the Θ-Net framework incorporated the said O-Net model (in its 1st node), a 120-epoch-trained O-Net model (for the 2nd node) and a 160-epoch-trained and transfer-learnt O-Net model (for the 3rd node), which is identical to that used for the DIC micrographs in Figure 3 previously. Also (as enunciated for the images in Figure 3), the **Source** (input) image was acquired via a 20X/0.40 Ph1 objective, while the **Expected** (ground truth) image was obtained with a 40X/0.60 Ph2 objective). *N.B.: This figure was sourced from Figure S13 of the accompanying Supplementary Materials. As with the Θ-Net-generated DIC micrographs in Figure 3 previously, the region encircled within the blue ellipse of the Θ-Net-generated images depicts an enhanced level of detail (as compared to its O-Net counterpart), supporting the proposed Θ-Net framework as a viable improvement over O-Net (for producing computational models to facilitate *in silico* ER). Here too (as with Figure 3 previously), the ANNA-PALM [23] model was utilized as an ImageJ extension for microtubule SR, while the 3D RCAN [26] model was trained over 250 epochs with 1972 steps per epoch and 2 residual groups. Contrast enhancement (in MS PowerPoint) was also applied to the Θ-Net-derived image, making it easier to discern the features.

Analyses of the images portrayed in Figure 6 (coupled with its corresponding Figure S13 in the accompanying Supplementary Materials) depict a relatively similar trend to that observed for Figure 3 (i.e., that Θ-Net-generated images more closely resemble the ground truth images as compared to O-Net-produced images), favoring the ‘*string of beads*’ *architecture* hypothesis (characteristic of Θ-Net) proposed in the present study to attain ER images in computational nanoscopy. Nonetheless, as with Figure 3 previously, there still exists room for improvement when comparing the effective resolution of the Θ-Net-generated images with the ground truth images, implying that more nodes/*beads* could putatively be added to the *‘string of beads’* Θ-Net architecture in a bid to improve ER images.

It would also be prudent to highlight at this juncture that the dilemma of network hallucination cannot be totally absolved from any of the model-generated images as depicted by the ROIs within the red and green ellipses in Figure S13, where some features evident within the **Expected** image were not detected in any of the DNN-processed images (including O-Net and Θ-Net). However, a closer inspection of the Θ-Net ER images does reveal a slight variation in pixel intensities at the points correlating to these features. Moreover, the ROI indicated by the green ellipse in the Θ-Net-generated image of Figure S13 seems to suggest potential artifacts caused by network hallucination within the yellow-colored channel, although the observed variations might also potentially arise as a consequence of phase variations in the sample instead.

**Figure 7:**
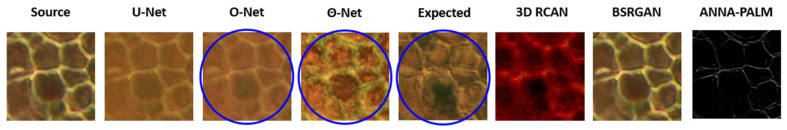
ER images obtained through several models, including those developed using O-Net [29] and Θ-Net. The O-Net model was trained over 101 epochs, while the Θ-Net model assimilated O-Net models trained over 120 epochs (for the 1st node), a 120-epoch-trained O-Net model (for the 2nd node) and a 160-epoch-trained and transfer-learnt O-Net model (for the 3rd node). Notice the increased resolution evident in the Θ-Net-generated image, which seemingly surpasses that generated via O-Net and even the **Expected** (ground truth) images, as highlighted within the blue ellipses. *N.B.: The **Source** image (input) was acquired via a 20X/0.40 Ph1 objective, while the **GT** (ground truth) image was obtained using a 40X/0.60 Ph2 objective. Images generated through models founded on other frameworks (namely 3D RCAN [26], BSRGAN [38] and ANNA-PALM [23]) were also included for comparison purposes (the ANNA-PALM [23] model developed for increasing the resolution of grayscale photomicrographs of microtubules was utilized as an extension within ImageJ 1.52n (NIH, USA), while the 3D RCAN [26] model was trained over 250 epochs with 1972 steps per epoch and 2 residual groups). Brightness enhancement (in MS PowerPoint) was applied to the Θ-Net-derived image, facilitating the discernment of the features. This figure was adapted from Figure S14 of the Supplementary Materials; further evaluation images are presented in Figure S14 for the interested reader).

Figure 7 (and its associated parent Figure S14) portrays how the Θ-Net models perform (in relation to other models, including O-Net and U-Net) with regards to increasing the resolution of PCM images for which they were *not* explicitly trained on (i.e., none of the models depicted here were trained with any of these images; hence, a verifiable deduction may be drawn in this respect). From these figures, we may observe that Θ-Net models generally provide increased spatial resolution (when compared to the other models, including U-Net and O-Net), although a closer inspection between the Θ-Net-derived images and the ground truth may reveal some slight differences between these two images. In this regard, one may be prompted to conclude that this is a consequence of network hallucination artifacts in Θ-Net, but it would be prudent to mention that this might not necessarily be so, as the Θ-Net model architecture adopts a transfer-learnt model (in its final O-Net mode) which incorporates similar features learnt differently from both DIC and PCM images. This allows Θ-Net to be more resilient to phase variations in samples when imaged across different phase-modulated microscopical modalities—an aspect which is not addressed through any one microscopical approach alone, thereby subjecting these imaging modalities to potential artifacts [such as the ‘halo’ effect (in PCM) or the pseudo-relief topography (observed in DIC)]. In the Θ-Net-generated images, however, these phase differences are converted into amplitude differences (when they fall within a certain range), allowing features to be resolved when these are often occluded by the ‘halo’ (or pseudo-relief) effects characteristic of PCM (and DIC microscopy).

**Figure 8:**
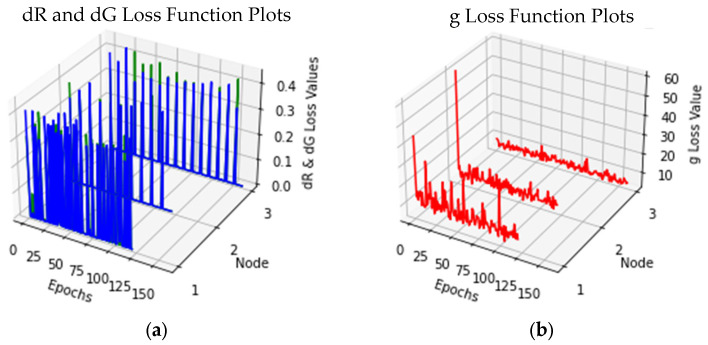
Loss function plots for Θ-Net models trained on PCM micrographs. (**a**) denotes the discriminator losses for real (dR) and generated (dG) samples, indicated by green and blue lines, respectively, while (**b**) represents the generator (g) loss (as red lines). As with Figure 5 previously, spikes present in the plots of the dR and dG loss functions indicate the start of a new training run (using the existing Python script), potentially implying the selection of a new random seed at the start of each run (model training for this dataset was also conducted intermittently).

As with the Θ-Net models utilized for increasing the resolution of DIC images earlier, we sought to plot the respective loss functions (dR, dG and g loss) for the various Θ-Net PCM models, as shown in Figure 8 below. Here too, a similar legend is used for the plots—blue is indicative of dG loss, green for dR loss and red for g loss.

In this respect, it would be vital to mention that when evaluating the performance of a generative adversarial network (GAN) such as that of Θ-Net, there is ***no*** objective loss function which can be used to conduct this [41]; hence, we chose to utilize the discriminator losses on the real and generated samples (as well as the generator loss) as key determinants for assessing Θ-Net performance. We conducted this for the Θ-Net models trained on *both* the DIC and PCM datasets (as depicted in Figure 5 and Figure 8, respectively). The formulae underlying these losses are indicated in the Discussion Section of this study.

### 3.3. Computation of Global and Local Image Metrics

**Figure 9:**
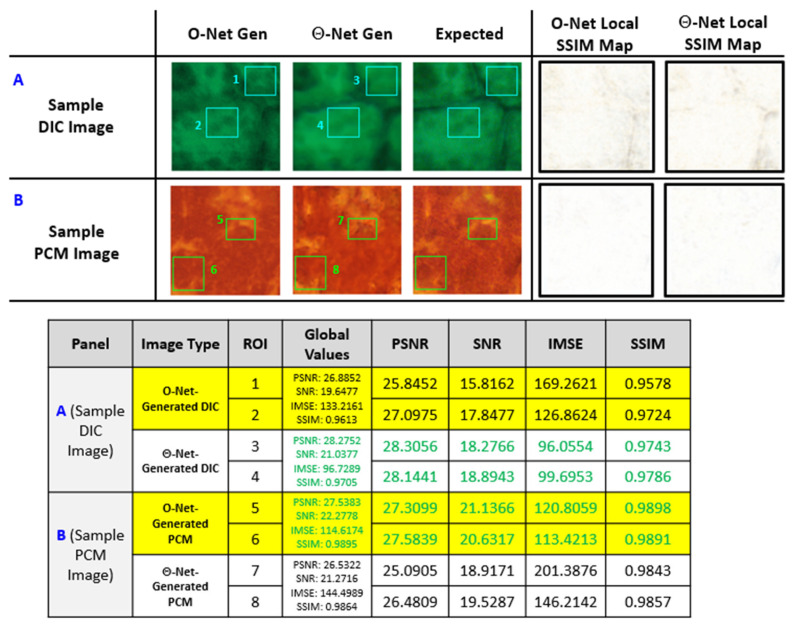
Sample O-Net- and Θ-Net-generated images from (**A**) DIC and (**B**) PCM micrographs. For this figure, a random sample was selected and assayed, as a means of exercising stringency when evaluating potential mismatches between Θ-Net and ground truth (**Expected**) images. Here, we observe that the local SSIM maps for both O-Net and Θ-Net appear to be generally white, which is indicative of a high correlation between the respective DNN-generated images and the ground truth (**Expected**) images [the local SSIM maps are based on individual pixel mismatches within an 11-by-11 neighborhood [42] and range from black (0) to white (1)]. From the table, it may be observed that Θ-Net generally surpasses O-Net when increasing the resolution of DIC micrographs, while the reverse holds true for PCM images (at least for the assayed images in this instance). Nonetheless (for the PCM images), the image metrics (such as PSNR and SSIM) for Θ-Net seem to closely approach those of O-Net (differing by <1% for SSIM scores but a slightly higher margin of <9% for PSNR scores), implying that Θ-Net models might be more susceptible to *local* variations in individual pixel values and seeking to convert these into discernible features, thereby resulting in an imposed ‘penalty’ and reduced SSIM scores (as these metrics may interpret these features as noise). Further evidence of this is provided through the IMSE metric, which clearly shows a marked increase for the Θ-Net-generated images (when compared with those from O-Net) for the PCM images (Panel **B**).

A further evaluation of the global and local image metrics (for specific ROIs in a separate subset of the validation images) was subsequently conducted, and the results are depicted in Figure 9 as follows.

From the findings in Figure 9, we may deduce that Θ-Net generally performs relatively well in increasing the resolution of phase-modulated micrographs (the images chosen in this context were randomly selected for validating both the O-Net and Θ-Net model performance). Despite this fact, regions where Θ-Net was noted to have underperformed (when compared to O-Net) were prominent in the PCM image dataset, although further inspection revealed that this reduced performance may be attributed to Θ-Net seeking to enhance the resolution of fine structural details within the **Source** image, resulting in lowered PSNR scores (since this metric is often used to penalize noise, while the latter may be reminiscent of *pseudo-noise*, as discussed in [29]). In this context, we would thus need to evaluate the performance of Θ-Net from the perspective of other metrics as well (such as SSIM), since these provide a holistic representation of Θ-Net as a DNN framework for *in silico* ER microscopy. Considering this perspective, we notice that the SSIM scores of Θ-Net-generated images closely approach those of O-Net (differing within 1%), implying that there is very little difference between the quality of Θ-Net- and O-Net-generated images for this (PCM) dataset

### 3.4. Image Denoising

**Figure 10:**
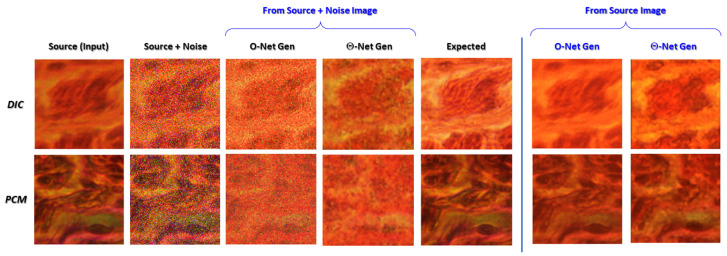
DIC and PCM photomicrographs infused with salt-and-pepper noise (noise density: 20%), which are labeled **Source + Noise**, and their corresponding ‘denoised’ images, as well as the ground truth (**Expected**) images utilized here. Here, we notice that Θ-Net models are relatively resilient to these noisy pixels, despite not being trained specifically to denoise images. This deduction is further corroborated by comparing these images against the Θ-Net-generated images in the absence of input noise (depicted within the violet ellipses). Nonetheless, it would be prudent to mention at this juncture that the Θ-Net models are still somewhat influenced by the noisy pixels in the image; hence, it would be preferred if the image denoising was performed *separately* prior to inputting the denoised images into Θ-Net for subsequent *in silico* ER.

To evaluate the efficacy of the proposed models in *denoising* input micrographs (even though the assayed models were **not** specifically trained for this purpose), we infused an artificial representation of noise into the **Source** images via a salt-and-pepper noise algorithm in MATLAB [43], with the image denoising consequently performed using the same trained models in Python. The results of this trial [including the corresponding **Source** and ground truth (**Expected**) images] are depicted in Figure 10 as follows.

### 3.5. Computational Complexity and Load

In addition to assessing the quality of ER images generated from each of the assayed architectures (in particular, Θ-Net), we also sought to compare the execution times for the afore-mentioned models, both as a means of quantifying their computational complexity and to determine the viable temporal resolution attainable by each of the models. The results following this analysis are presented in Table 1 below.

From the findings tabulated in Table 1, we may observe that the average execution times of Θ-Net (a DNN architecture comprising three O-Net nodes strung together in the present context) expectably exceed those of O-Net, although this increase does not appear to be proportional to the node count of the Θ-Net framework used. Instead, the execution times seem to be highly dependent on the type of GPU used, ranging from a maximum multiplier of ~9.2X (for the NVIDIA Tesla K80 GPU system) to a low of ~1.4X (for the NVIDIA RTX 3090 GPU system). As GPUs are known to excel in parallel computationally intensive tasks, this might suggest that the individual O-Net nodes are being executed in a parallel (rather than a sequential) fashion.

## 4. Discussion

The results depicted in this study exhibit the significant potential of Θ-Net in attaining computational phase-modulated nanoscopy. Here, it may be observed that Θ-Net (as with O-Net in [29]) can enhance the resolution of both DIC and PCM micrographs while avoiding the formation of potential artifacts characteristic of these imaging modalities (namely the pseudo-relief effects in DIC microscopy [44] and the halo/shading-off effects of PCM [45]). In this regard, we surmise that the Θ-Net-based models likely conduct this (i.e., ER imaging) via a mapping function to reduce the PSF/OTF of the optical system (akin to that for O-Net as described in [29]), resulting in a hypothetical PSF which (when convolved with the ground truth representation) produces an ER image of the specimen in question (further details on this are expounded in [29] for the interested reader). However, Θ-Net conducts this through the *repeated functional mapping* of the acquired/generated PSF-convolved input image, which may be generally described by Equations (1) and (2) below:

**Learning Phase**

(1) $$\left(f⊛g\right)\;\underset{n\;times}{\underbrace{\overset{j_{1}}{\to }\ldots \;\overset{j_{n}}{\to }}}\;\left(f_{n}⊛g\right)\;\sim \;\left(h⊛g\right)$$

where *f*_1_ refers to the PSF of the optical system when using the 20X/0.4 Ph1 objective, *g* is the ground truth image of the specimen and *h* is the PSF of the optical system (when using the 40X/0.6 Ph2 objective). Here, *f*_n_ refers to the *generated* ‘PSF’ of the image when (*f* ⊛ *g*) is mapped under *j*_n_ [where $\underset{n\to \infty }{\lim}\left(f_{n}\right)=h$]. Like O-Net [29], the Θ-Net models thus attempt to learn the mapping function *j*_1…n_ across *n* nodes (for which Θ-Net is defined) and (upon sufficiently learning this) deploy *j*_1…n_ for ER images as follows:

**Resolution Enhancement Phase**

(2) $$\left(f⊛g\right)\;\;\;\underset{n\;times}{\underbrace{\overset{j_{1}}{\to }\ldots \;\overset{j_{n}}{\to }}}\;\;\underset{\begin{array}{c} \mathrm{enhanced}\;\mathrm{resolution}\\ \mathrm{image} \end{array}}{\underbrace{\left(f_{n}⊛g\right)}}$$

Nonetheless, a key aspect of our present study remains to highlight the feasibility of models adopting the Θ-Net framework over their predecessors employing the O-Net framework (as described in [29]) for *in silico* label-free ER microscopy—evidence for this being presented in Figure 3, Figure 4, Figure 6 and Figure 7 (and their corresponding Supplementary Figures S11–14). As previously indicated, Θ-Net (in the present study) employs a *triple*-node architecture (each node being an O-Net) with a specialized *transfer-learnt* O-Net model for the last node (the said node being trained on *both* the DIC and PCM datasets) (see Figure 2 for details). Here, the transfer learning process clearly aids in enhancing the ER capabilities of the Θ-Net models, putatively by allowing for a *transfer* of learnt phase–amplitude translations across two principally similar (yet methodologically different) microimaging modalities (i.e., DIC and PCM) to augment the trained models’ abilities in detecting and translating these phase variations to the observable feature space. The empirical validation of this statement may be derived from Figure 4 and Figure 7, where the granularity of some structural features (such as the surface of the cell wall) becomes distinctly visible upon processing with the Θ-Net models (as compared to the O-Net models), closely corroborating one’s prior knowledge of such ultrastructural features (see [46] as an example for details).

Of particular importance to some would be the questions of why a different number of epochs was used for each of the O-Net nodes and what the significance of the chosen number of nodes in the Θ-Net architecture is. Here, it would be imperative to highlight that both of these factors were empirically determined for the acquired dataset—to minimize the model training duration while seeking to attain the optimal image accuracy (as described in Figure 4 and Figure 7). In addition, one may also seek to question our choice of activation functions used in this study (namely Swish and GELU) and why more popular activation functions (such as ReLU or its leaky variant) were not utilized instead. To comprehend the rationale behind this, we would first need to take a step back and realize that elucidating the PSF of an optical system (such as a compound optical microscope) is a complex, non-linear problem, and the use of different activation functions (such as Swish and GELU) would potentially aid in mitigating some of the PSF variations introduced by sample inhomogeneities at different points in the specimen, thereby allowing the network to more closely approximate the convolution of the ground truth signal with the optical PSF at these different regions (as the light rays pass through the optical train of the microscope). Due to the highly complex nature of this problem [it is not possible to simply use a single formula to deduce the optical PSF and apply it via a deconvolution algorithm to the entire image (as is often the case for most computational deconvolution algorithms, whether blind or non-blind)], we postulate that the use of different activation functions for the discriminator and the generator networks (in particular, Swish and GELU which closely approximate the intensity distribution as introduced by the Airy function of the PSF) would allow for a convergence between these two networks into a more optimal solution. If only a single activation function (such as ReLU or Leaky ReLU) were used, these might not be able to sufficiently account for the spatiotemporal variations in the PSF, resulting in the production of output images which might not contain the required level of detail for SR images. It is, however, possible that only the use of ReLU or GELU might suffice (to a certain extent), although this approach was not validated in the present study.

From a computational perspective, the execution times of Θ-Net seem to be viable as well, generally not exceeding 1s in current advanced GPU systems, making it viable where processes having a temporal resolution exceeding 1s are generally encountered. Moreover, the loss function plots of the assayed Θ-Net models (for both DIC and PCM) suggest that the models were optimally trained, with the discriminator losses for both the real and generated samples (indicated by dR and dG, respectively) generally approaching 0 for all three nodes. The equations underpinning the computed losses [dR, dG and the generator loss (g)] may be mathematically defined as follows:

For the discriminator losses (i.e., both dR and dG), the **binary cross-entropy** loss (or log loss) *ε* was utilized, which may be expressed as below (from [29,47]):

(3) $$\varepsilon =\frac{\sum_{i=1}^{n}\left[\left(b_{i}-1\right)\log\left(1-{\hat{b}}_{i}\right)-b_{i}\log{\hat{b}}_{i}\right]}{n}$$

where *b_i_* is the label, and ${\hat{b}}_{i}$ is the probability of *b*_i_ = 1 (derived from [47,48] and described in [29]).

In contrast, for computing the generator (g) loss, both *ε* and the **mean absolute error** (MAE)/l_1_ loss were used, the latter being computed as follows (from [29,49]):

(4) $$\mathrm{MAE}\;(l_{1})\;\mathrm{loss}=\frac{\sum_{i=1}^{n}\left|a_{i}-b_{i}\right|}{n}$$

where *n* is the number of pixels in the image, and *a_i_* and *b_i_* refer to the target and the estimated values of the assayed parameter [e.g., pixel RGB (or HSL) intensities at pixel *i*, respectively].

Compounded together, the overall generator loss G may thus be expressed by the following equation (from [29]):

Total generator loss, G = ε + (λ · l_1_) (5)

where λ = 100 [50] (to minimize potential artifacts, as described in [30]). ε and the MAE were utilized as viable loss functions for evaluating model training in this respect as they provide viable indications on how closely the generated images resembled the ground truth (**Expected**) image under differing representations (the MAE evaluates this from a linear perspective, while ε adopts a logarithmic standpoint).

On a separate note, the empirical verification of the image denoising capabilities of the Θ-Net models suggests that these models are relatively resilient to salt-and-pepper noise [43] present in the images, although there does exist a slight possibility of the models confounding this noise with sub-microscopic features which require a modulation transfer function (MTF) greater than that afforded by the microscope’s optical train [51]. Evidence for this may be drawn from Figure 10, where some features encircled within the blue ellipse of the DIC micrograph depict an attempt by the said model to translate some of the noise into ‘ER features’ (a characteristic generally absent in traditional O-Net-based models). This finding thus portrays the need for Θ-Net users to denoise the input images separately, prior to increasing the resolution of them via the proposed models (to avoid introducing noise-based artifacts into the image). Despite this fact (and as previously already highlighted in the caption for Figure 10), the Θ-Net models perform significantly better in removing noisy pixels from the image (when compared to O-Net), although they were *not* specifically trained for this purpose (i.e., to conduct image denoising). This thus demonstrates a greater propensity of Θ-Net in image denoising applications, though potentially at the expense of incurring network hallucination artifacts.

Retrospectively, a further evaluation of the image quality metrics used (namely PSNR, SNR, IMSE and SSIM) seemingly suggests otherwise, with Θ-Net models generally surpassing O-Net model performance when increasing the resolution of DIC micrographs, although the opposite occurs for the PCM images. Nonetheless, this assessment proves contrary to the visual discrimination of the features present in the Θ-Net-generated PCM micrographs (when compared with their O-Net counterparts), casting doubt on the veracity of these metrics for quantifying the ER capabilities of a DNN algorithm developed for this purpose. This concern was also surfaced previously in [29], where a suggestion recommending the future development of a suitable metric for quantifying the “super-resolution” quality of an image was proposed, although this metric would probably be difficult to validate, as it would have to consider the illumination type and relative intensity of the light source (in addition to the specimen clarity and mounting procedure) being employed, amongst others. Moreover, Nyquist sampling criteria would also have to be satisfied [52] to alleviate potential network hallucination due to under-sampling operations which may result in sample noise being confounded as *pseudo*-noise [18].

It would also be noteworthy to mention that the 3D RCAN [26] models used for comparison against the evaluated O-Net and Θ-Net models in this study were developed based on an RGB image spectrally isolated into a 3-channel grayscale image stack within ImageJ 1.52n (NIH, USA), prior to training and executing the models on these images. This was because the 3D RCAN framework [26] was specifically developed to enhance the resolution of grayscale fluorescent micrographs in a 3D Z-stack, although our training images acquired were 2D 24-bit RGB images. The output image stack was subsequently re-merged in ImageJ into a single RGB image.

### 4.1. The Potential Advantages and Limitations of the Present Study

#### 4.1.1. The Limitations of the Current Θ-Net Architecture

A prominent limitation in the current study (which is common to all DNN architectures) refers to the data source which was used to train the models. In the present context, our proposed Θ-Net models were trained on data acquired using a Leica DM4000M microscope with a RisingCam^®^ CMOS camera. Should the models of the microscope and camera used to acquire the test images be different, the models would need to be retrained to compensate for the variations in the PSF experienced in the optical train in this respect. For this reason, readers who intend to use our supplied Θ-Net models for their own PCM and/or DIC photomicrographs would have to ensure that an identical microscope and camera setup is used for image acquisition (as highlighted in the present study), or a retraining of the models might be required.

On a separate note, we also *only* demonstrated the use of Θ-Net in increasing the resolution of PCM and DIC images—two highly popular phase-modulated optical microscopical approaches. Here, and as was also highlighted in [29], numerous other variants of phase microscopy techniques exist, notably Hoffman modulation contrast [53] and oblique illumination [54], as well as more recently developed (yet increasingly popular) quantitative phase imaging modalities [such as digital holographic microscopy (DHM) [55], amongst others]. As we did not verify the applicability of Θ-Net in increasing the resolution of images gleaned from each of these techniques, we are unable to hypothesize the putative performance of Θ-Net in this respect. It would also be noteworthy to highlight here that these techniques (i.e., PCM and DIC microscopy) are *semi-quantitative* approaches for representing phase variations in the sample and cannot be used to precisely *quantify* the phasic information due to the non-linear (and thus non-invertible) relationship between the phase and amplitude in different regions of the sample [56]. However, in our current study, we did not seek to specifically quantify these phase differences but simply use the said models to translate these phase variations in the sample (caused by the different PSFs in the optical train) into amplitude (brightness) differences for ER images.

#### 4.1.2. The Advantages of the Current Θ-Net Architecture

As described in the captions for Figure 5 and Figure 8 previously, the training of the Θ-Net models in the present study was performed *intermittently*, which resulted in the spikes observed in the loss function plots for the discriminator network (see Figure 5 and Figure 8 for details). Here (and as was also demonstrated in [29,40]), intermittent model training generally results in more accurate models, visualized as the similarity between the generated (output) images and the ground truth (**Expected**) image datasets. This phenomenon may be postulated to be due to the minimization of the *trapping* of the loss function within local minima in the global error landscape, an aspect which we also surmised in [40].

Holistically, from the results gleaned and presented thus far, we may postulate that the proposed Θ-Net architecture poses significant usability in increasing the resolution of images acquired via PCM and DIC microscopy (two widely utilized label-free diascopic imaging techniques in the optical microscopical space today). In this respect, models developed using Θ-Net hold significant promise in future label-free optical nanoscopical applications, facilitating numerous potential extrapolations of engineering applications utilized in the industry today and moving towards the future (i.e., Industry 4.0 and beyond). Nonetheless, it would also be noteworthy at this juncture to address the perception that the Abbe diffraction limit *cannot* be surpassed through blind *in silico* approaches, as this information was effectively lost due to the physical limitations imposed by the imaging system. Proponents of this view may also suggest that a black-box model (such as a trained DNN) without any priors from the imaging physics or from the sample can only show the learnt features from the data (and nothing more). However, this perception may be challenged by the fact that there are numerous optical approaches (such as [57]) which demonstrated the circumvention of the Abbe limit, while our present study provides an alternative (non-optical) approach to achieving optical non-fluorescent nanoscopy (via phase-modulated *in silico* ER). All of these studies thus point to the fact that the information encoded by such high spatial frequencies is *not* entirely lost in the optical train (as might be perceived by these individuals), but it is simply *obscured* by other dominant (lower spatial frequency) impulse responses. Moreover, based on our current findings, we can somewhat propose the ability of Θ-Net as a potential means of recovering some of this occluded high spatial frequency information, in a bid to achieve *in silico* ER so as to attain computational phase-modulated nanoscopy in the near future. This might thus be utilized to facilitate further developments in fields as diverse as *bioengineering* (for the synthesis of nano-scaffolds to direct proteins to specific sites for proper folding and functional deployment), *medical imaging* (for detecting and identifying nascent tumorigenic cells), *material sciences and electronics engineering* (for quality analysis and cost-friendly nanoscale defect inspection of semiconductor wafers and in nanolithography) and *optical engineering and photonics* applications (to facilitate the research and development of new optical modulators, lasers and diodes), where microscopes remain indispensable tools for both the diagnosis and quality inspection/analysis of parts (amongst others).

## 5. Conclusions

In the current study, the performance of our newly developed DNN architecture (Θ-Net) was assessed against other popular state-of-the-art DNN architectures (O-Net [29], 3D RCAN [26], ANNA-PALM [23] and BSRGAN [38]) with respect to its accuracy in increasing the resolution of PCM and DIC micrographs computationally. Our models depict a relatively high level of accuracy, generating images which come close to the ground truth (**Expected**) images. Notably, ER images generated by the Θ-Net models of untrained images (Figure 4 and Figure 7, as well as Figures S12 and S14) closely agree with prior knowledge of cellular ultrastructure, exemplifying the usability of Θ-Net in this context. In this regard, one may be led to recommend Θ-Net-derived models for increasing the resolution of PCM and DIC images, although the other compared models (such as ANNA-PALM [23] and 3D RCAN [26]) might perform relatively well for other imaging modalities (such as epi-fluorescence microscopy stacks) (it would be noteworthy to mention here though that these models were not specifically trained for increasing the resolution of phase-modulated microscopical images, such as those acquired via DIC and PCM, as in the present study).

Despite this fact, there currently exists *no* viable image SR metric which may be used to objectively quantify the performance of a DNN model in resolving images. This dilemma is further exacerbated through the inappropriate use of popular image metrics (such as PSNR, SSIM and IMSE, amongst others) for this purpose in past studies exploring computational SR imaging. A probable resolution of this issue may thus lie in the development of such a metric specifically for ER/SR imaging, although this would have to consider multiple factors such as the effect of different illumination intensities, camera exposure and gain in the acquired image. A further treatment of this issue is hence presented in [29] for the interested reader.

Finally, we would also like to highlight that numerous recent cutting-edge research studies have demonstrated the potential of DNNs in various medical imaging approaches, ranging from ultrasound localization microscopy ([58]) to computer-aided detection ([59]) and MRI-CT ([60]), thereby providing an avenue for the potential applicability of our proposed Θ-Net architecture in a similar regard. Similarly, other far-ranging potential applications of Θ-Net may also be surmised in LiDAR–hyperspectral imaging and/or remote sensing applications (e.g., drone imaging), although the use of Θ-Net models for the latter might be subject to other potential constraints as well (as described at [61]) while also necessitating model retraining in this respect.

## Figures and Tables

**Table 1:** Execution times (in seconds) indicated for each of the models trained under the assayed frameworks (i.e., O-Net and Θ-Net) in the present study. As Θ-Net comprises 3 nodes (each of which is an O-Net model), the implementation of Θ-Net for image post-processing is expected to take ~thrice as long as that of O-Net, although this is a non-linear relationship, being very much dependent on GPU capabilities.

Architecture	CPU	GPU	RAM	Storage	Average Execution Time
**O-Net**	Intel^®^ Core™ i5-7200U CPU	NVIDIA GeForce^®^ 920MX	20 GB	1 TB SSD	11.132 s (DIC)/11.463 s (PCM)
	2 x Intel^®^ Xeon^®^ Platinum 8170 (56C/112T)	NVIDIA Tesla K80	128 GB	500 GB SSD	2.437 s (DIC)/2.132 s (PCM)
	Intel^®^ Core™ i9-10920X	NVIDIA GeForce RTX^TM^ 3090	128 GB	512 GB SSD	0.422 s (DIC)/0.297 s (PCM)
**Θ-Net**	Intel^®^ Core™ i5-7200U CPU	NVIDIA GeForce^®^ 920MX	20 GB	1 TB SSD	51.756 s (DIC)/40.291 s (PCM)
	2 x Intel^®^ Xeon^®^ Platinum 8170 (56C/112T)	NVIDIA Tesla K80	128 GB	500 GB SSD	14.89 s (DIC)/19.613 s (PCM)
	Intel^®^ Core™ i9-10920X	NVIDIA GeForce RTX^TM^ 3090	128 GB	512 GB SSD	0.593 s (DIC)/0.703 s (PCM)

## Data Availability

The data utilized for this study (figures, models and codes) may be downloaded at https://drive.google.com/file/d/1J0HYPE6-tOJOJ9F_fkifzomQ6oFxJ-Mc/view?usp=sharing (accessed on 22 September 2024).

## Supplementary Materials

The following supporting information can be downloaded at: https://www.mdpi.com/article/10.3390/s24196248/s1, Figure S1: Diagram illustrating the model parameters for the O-Net GAN; Figure S2: Diagram illustrating the model parameters for the O-Net GAN discriminator (A) & generator (B) respectively; Figure S3: Diagram illustrating the model parameters for the 2nd node of the Θ-Net GAN; Figure S4: Diagram illustrating the model parameters for the 2nd node Θ-Net GAN discriminator (A) & generator (B) respectively; Figure S5: Diagram illustrating the model parameters for the 3rd node of the Θ-Net GAN; Figure S6: Diagram illustrating the model parameters for the 3rd node Θ-Net GAN discriminator (A) & generator (B) respectively; Figure S7: Diagram illustrating the model parameters for the O-Net GAN; Figure S8: Diagram illustrating the model parameters for the O-Net GAN discriminator (A) & generator (B) respectively; Figure S9: Diagram illustrating the model parameters for the 2nd node of the Θ-Net GAN; Figure S10: Diagram illustrating the model parameters for the 2nd node Θ-Net GAN discriminator (A) & generator (B) respectively; Figure S11: Figure illustrating the model-generated images for the DIC training images dataset presented in this study; Figure S12: Figure illustrating the model-generated images for the DIC validation dataset presented in this study; Figure S13: Figure illustrating the model-generated images for the PCM training images dataset presented in this study; Figure S14: Figure illustrating the model-generated images for the PCM validation dataset presented in this study; Table S1: Equations considered for the activation functions; Table S2: Equations considered for the optimizer function. References [62,63,64,65,66,67,68] are cited in Supplementary Materials.
