# Supplementary material for: Θ-Net: A Deep Neural Network Architecture for the Resolution Enhancement of Phase-Modulated Optical Micrographs In Silico
Source: Sensors (Basel). 2024 Sep 26;24(19):6248. doi: 10.3390/s24196248 (PMC11478931; doi:10.3390/s24196248)
Supplement: Supplementary file 1 [file sensors-24-06248-s001.zip › sensors-3208908-supplementary.pdf]

Supplementary Information

1. Technical parameters defined for O-Net & Θ-Net

The architectures for both the O-Net & Θ-Net GANs, as well as the various parameters used for different aspects of these frameworks to super-resolve DIC & phase contrast microscopy (PCM) images in the present study, are illustrated in the following sub-sections:

i. O-Net Framework for DIC super-resolution (SR)

The O-Net Pix2Pix GAN to perform DIC SR is depicted in the following diagram:

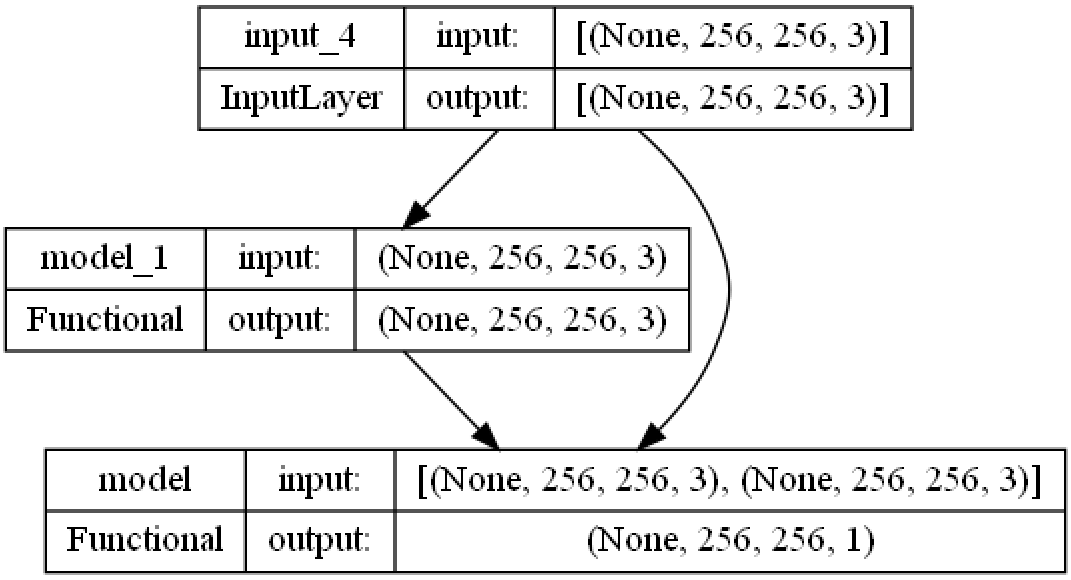

Supplementary Figure S1: Diagram illustrating the model parameters for the O-Net GAN.

A schematic of the discriminator & generator architectures are provided as follows:

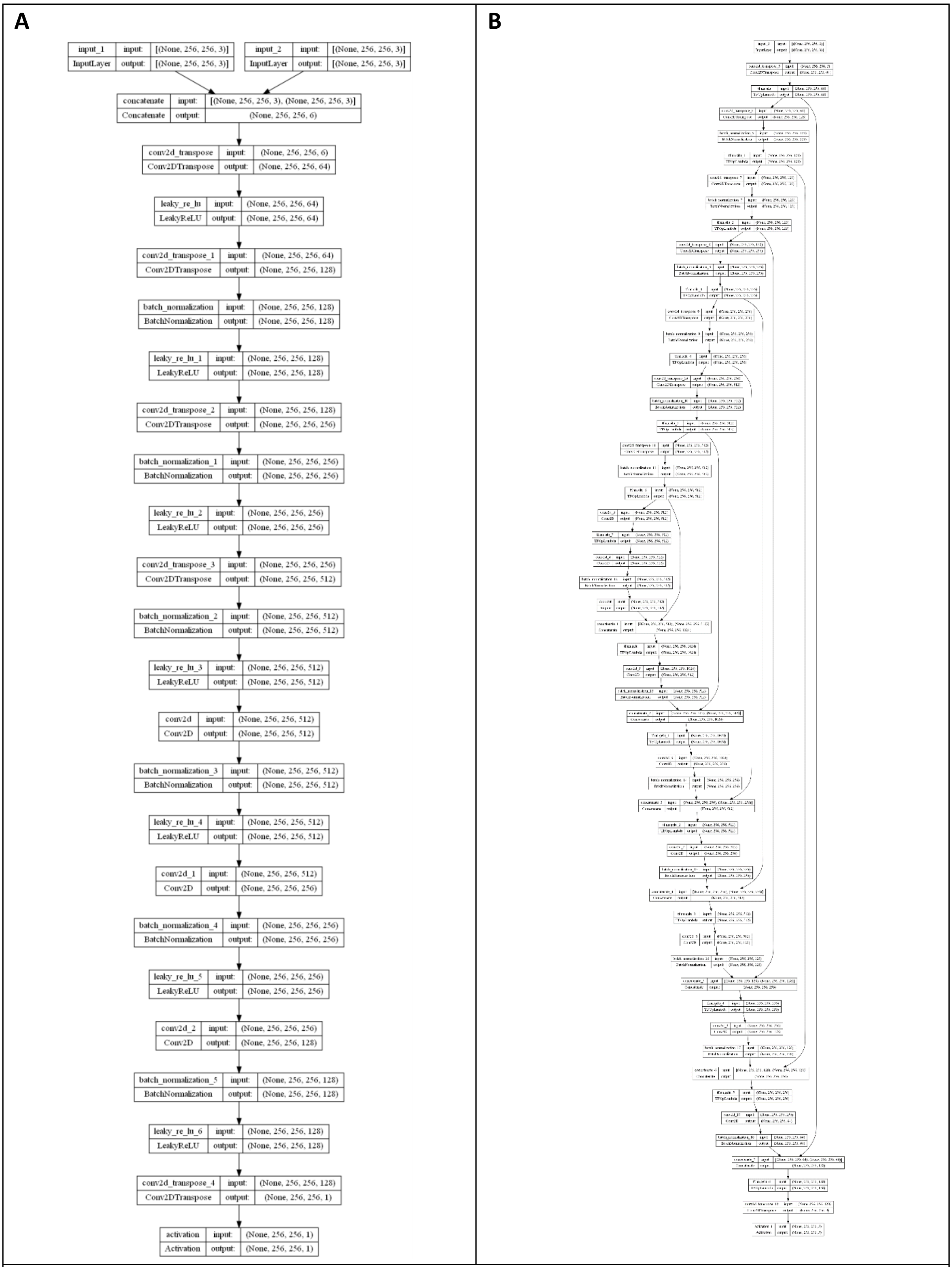

Supplementary Figure S2: Diagram illustrating the model parameters for the O-Net GAN discriminator (**A**) & generator (**B**) respectively.

ii. Θ-Net Framework for DIC SR

The Θ-Net Pix2Pix GAN used to perform DIC SR in the present study is based on a triple-node string – each node comprising of an O-Net architecture. For the current context, the 1st node of the Θ-Net framework used for DIC SR is identical to that of the O-Net models described in sub-section (i) previously. The 2nd & 3rd nodes of the Θ-Net structure may be described as follows:

<u>For the 2nd Node:</u>

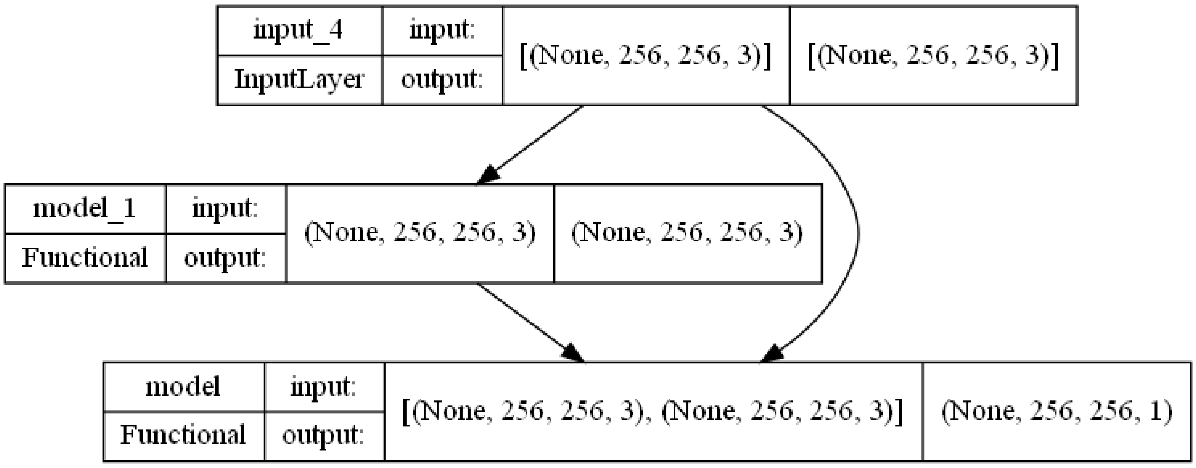

Supplementary Figure S3: Diagram illustrating the model parameters for the 2nd node of the Θ-Net GAN.

As previously, the discriminator & generator architectures may be depicted as follows:

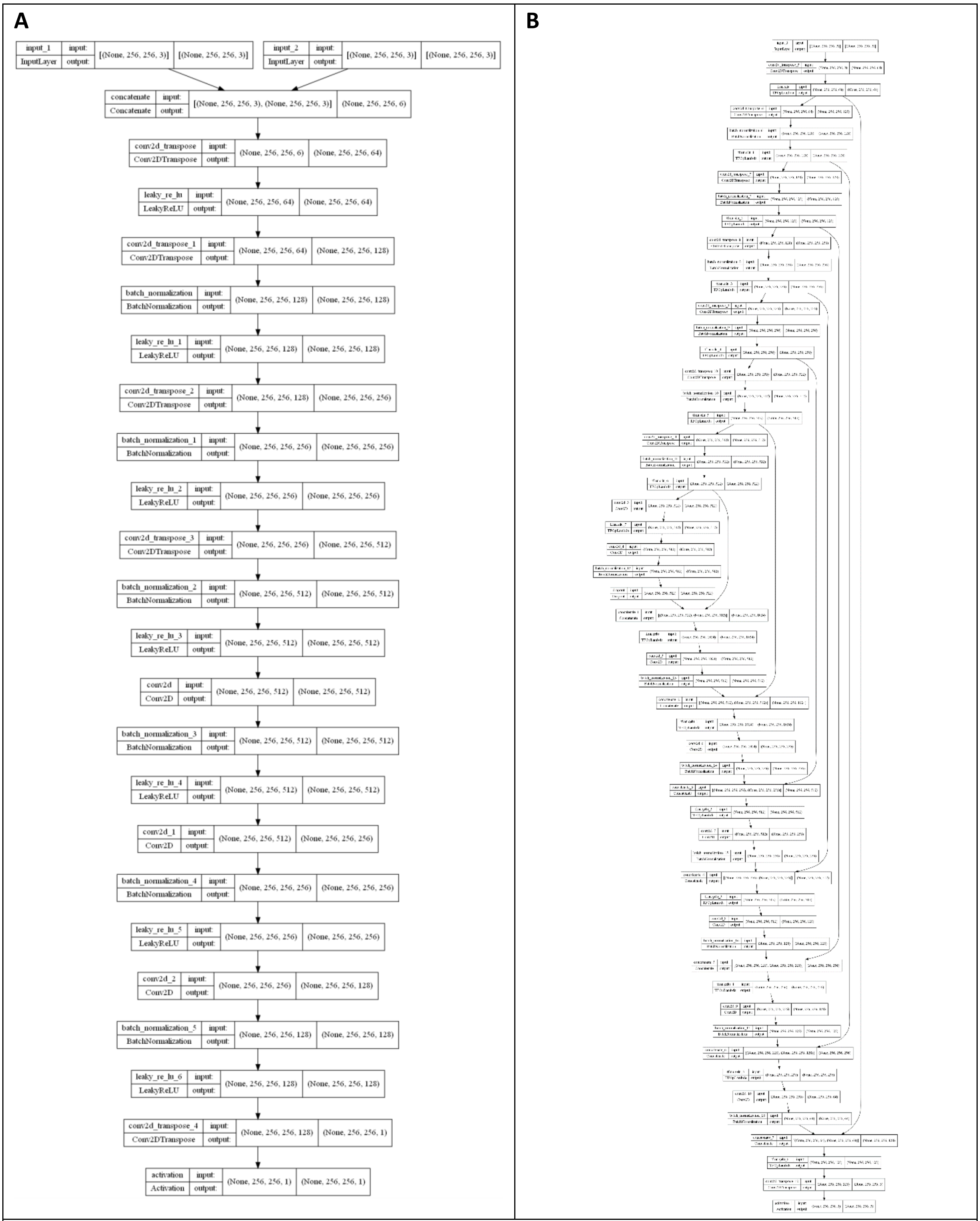

Supplementary Figure S4: Diagram illustrating the model parameters for the 2nd node Θ-Net GAN discriminator (**A**) & generator (**B**) respectively.

For the 3rd Node:

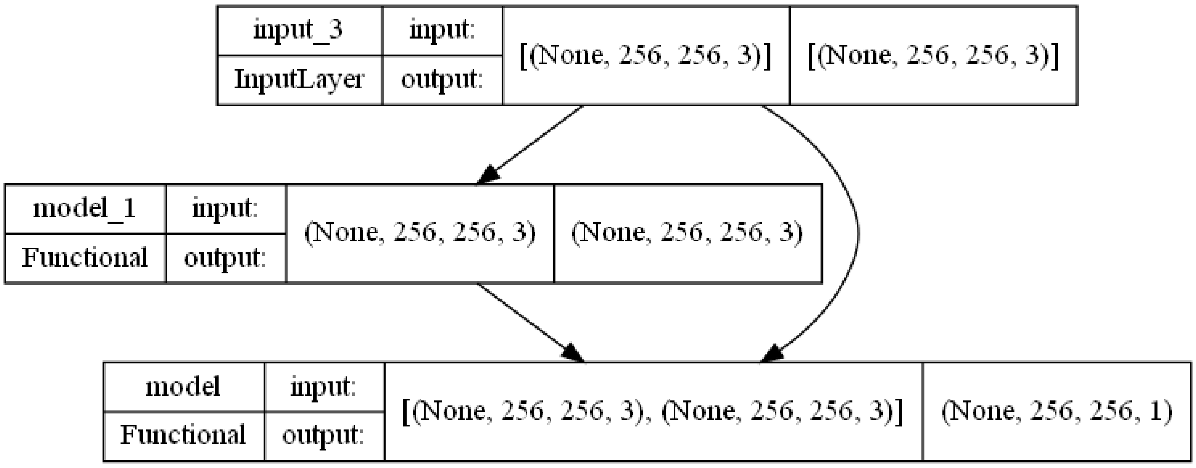

Supplementary Figure S5: Diagram illustrating the model parameters for the 3rd node of the Θ-Net GAN.

In the 3rd node, the discriminator & generator architectures may be depicted as follows:

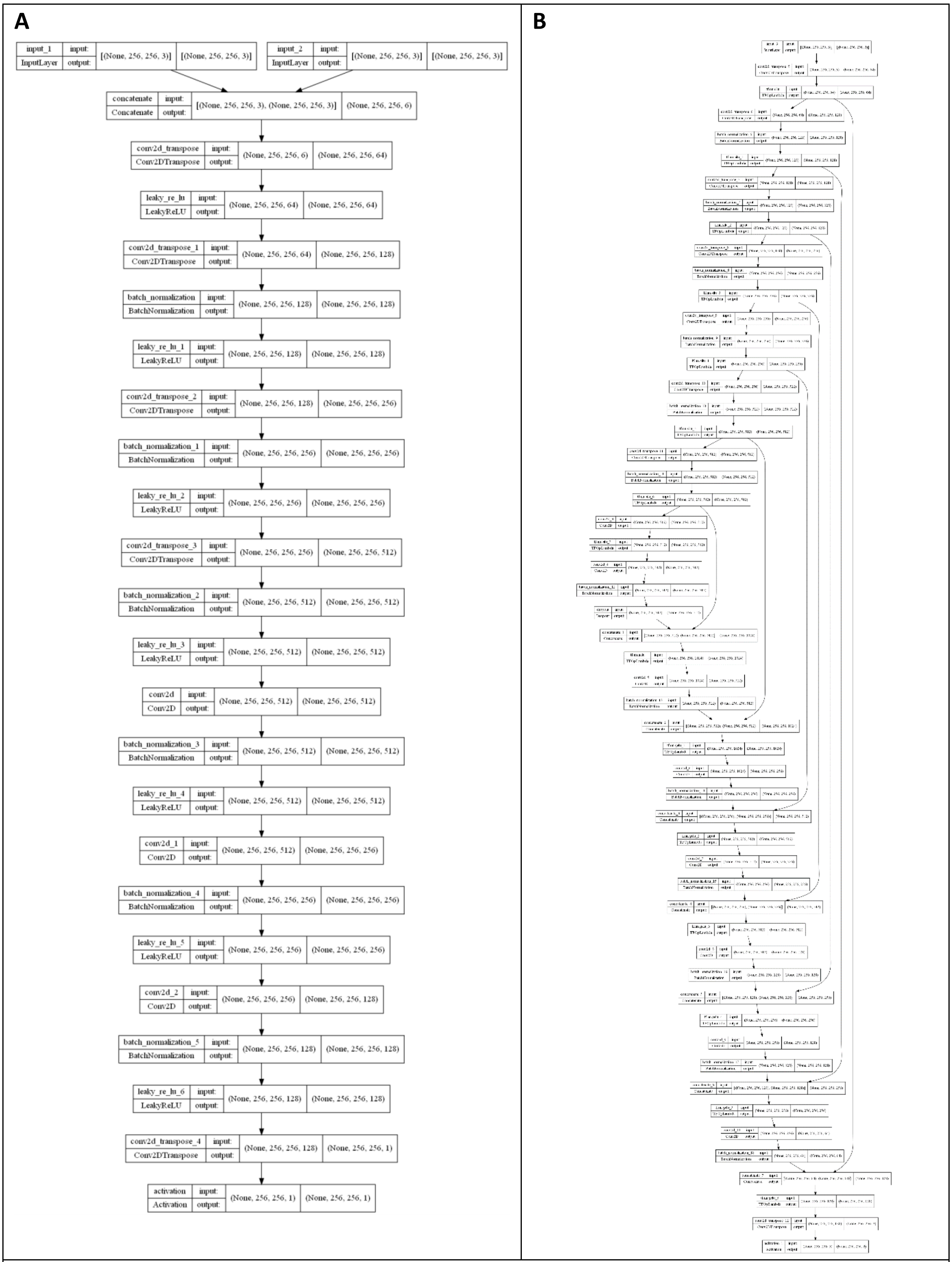

Supplementary Figure S6: Diagram illustrating the model parameters for the 3rd node Θ-Net GAN discriminator (**A**) & generator (**B**) respectively.

iii. O-Net Framework for PCM SR

The O-Net Pix2Pix GAN to perform PCM SR is depicted in the following diagram:

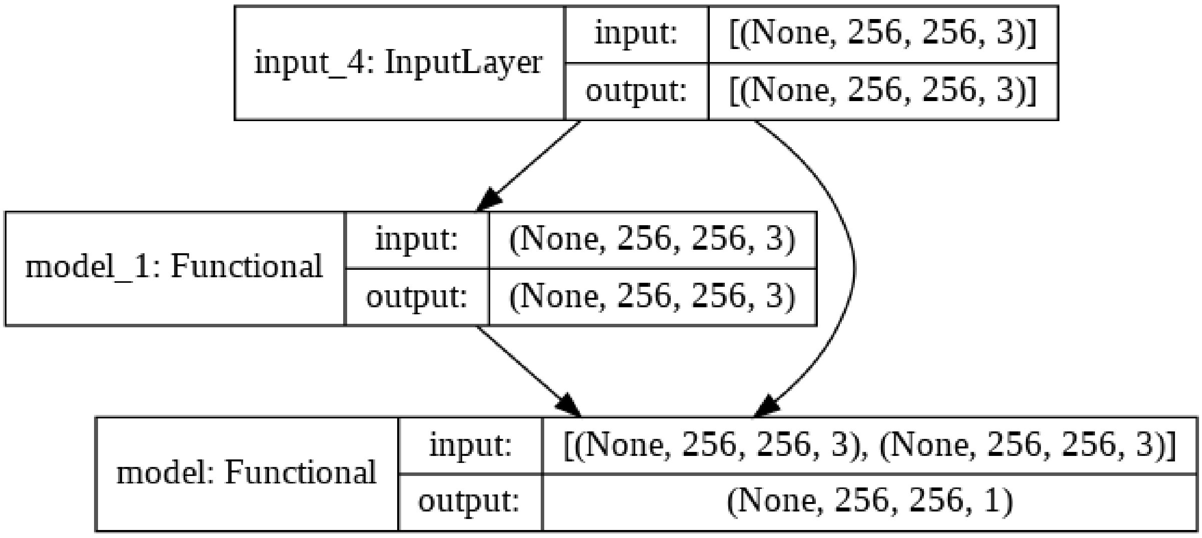

Supplementary Figure S7: Diagram illustrating the model parameters for the O-Net GAN.

A schematic of the discriminator & generator architectures are provided as follows:

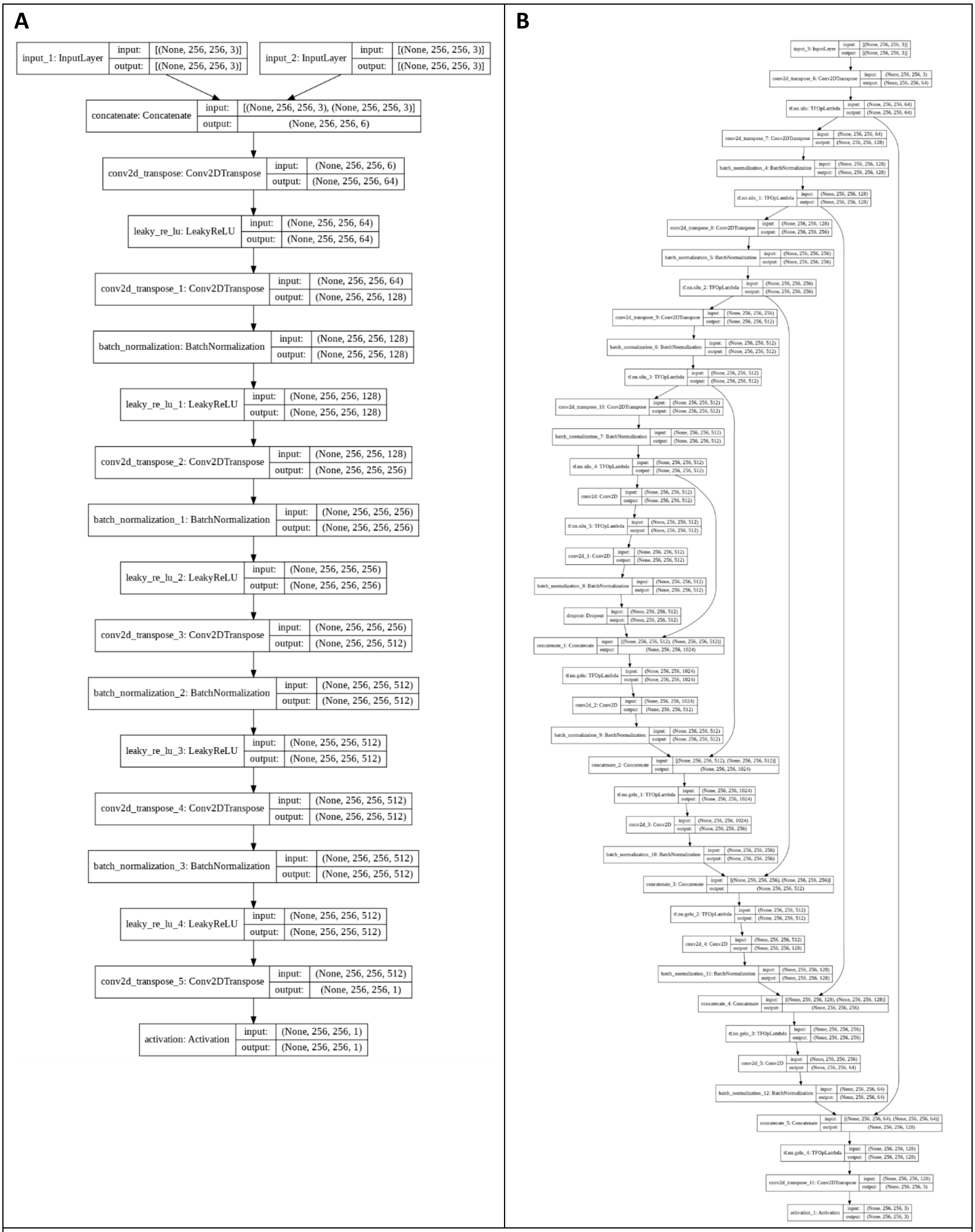

Supplementary Figure S8: Diagram illustrating the model parameters for the O-Net GAN discriminator (**A**) & generator (**B**) respectively.

iv. Θ-Net Framework for PCM SR

As with the Θ-Net architecture used for DIC SR in sub-section (ii) previously, the Θ-Net Pix2Pix GAN utilized for PCM SR in the current study is also based on a triple-node string, with each node consisting of an O-Net model. However, in the present context, the 1st node of the Θ-Net framework used for PCM SR is identical to that of the O-Net model described in sub-section (iii) previously. In addition, the 3rd node of the Θ-Net structure also employs an identical structure to that of the 3rd node of the Θ-Net framework used for DIC SR [highlighted in subsection (ii) previously]. Thus, only the 2nd node of the Θ-Net string used for PCM SR is unique to this pipeline, with the structure of the said models for this node being described as follows:

<u>For the 2nd Node:</u>

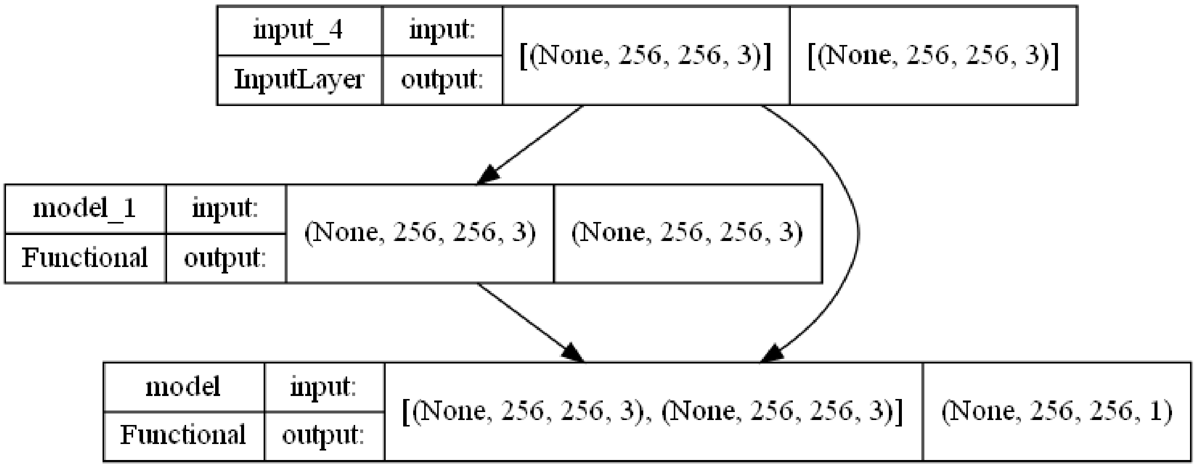

Supplementary Figure S9: Diagram illustrating the model parameters for the 2nd node of the Θ-Net GAN.

As previously, the discriminator & generator architectures may be depicted as follows:

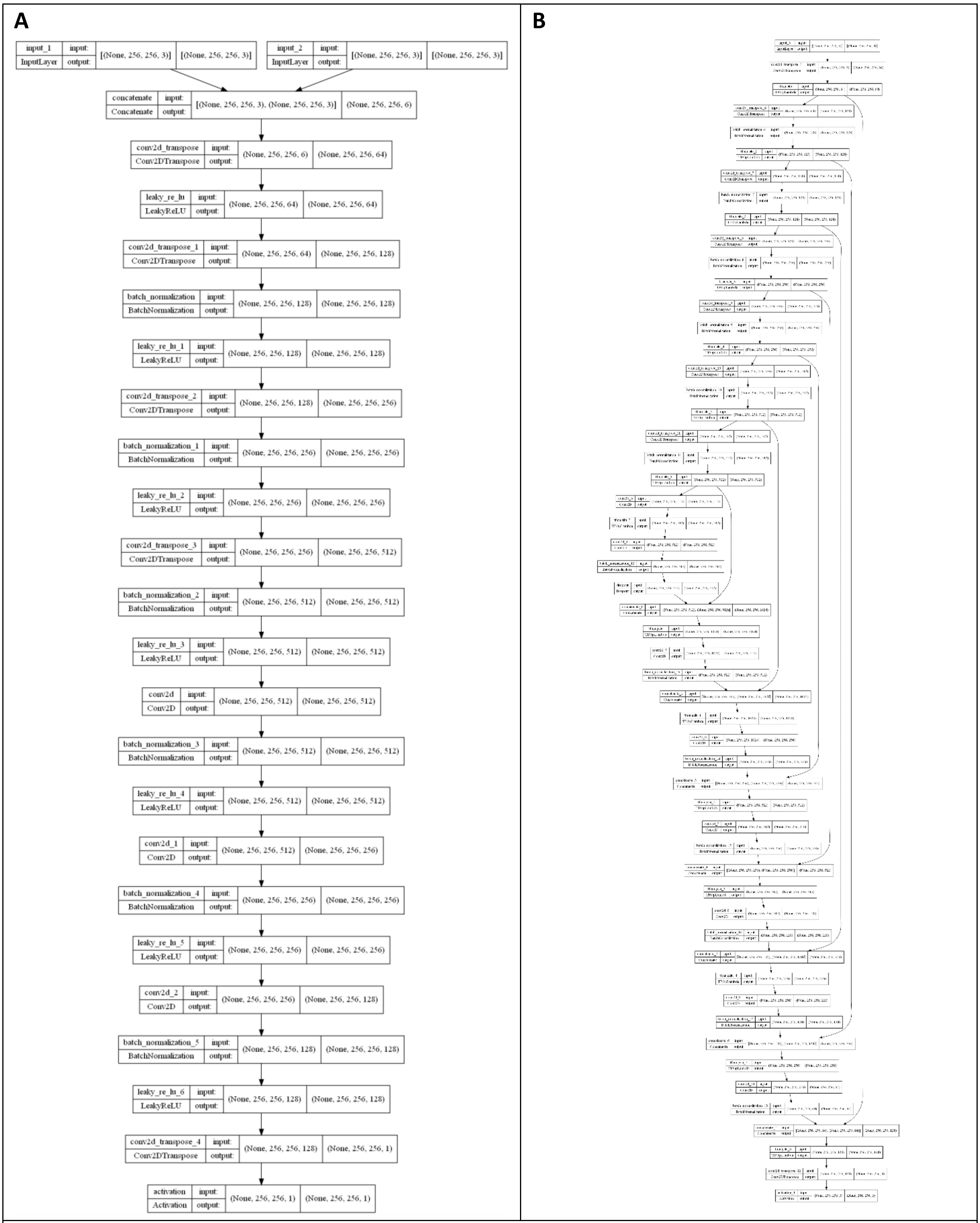

ⲟ **Supplementary Figure S10**: Diagram illustrating the model parameters for the 2nd node Θ-Net GAN discriminator (**A**) & generator (**B**) respectively.

2. Equations considered for the activation functions

5 different activation functions were used in the O-Net & Θ-Net networks for the present study.

These activation functions are as follows:

Activation Function	Equation	Source
**Leaky ReLU**	$y = \begin{cases} \alpha \cdot x & x < 0 \\ x & x \geq 0 \end{cases}$ ---------- (S1)	[62]
**Sigmoid**	$y = \frac{1}{1+e^{-x}}$ ------------------------------ (S2)	[63]
**Tanh (Hyperbolic tangent)**	$y = \tanh(x) = \frac{e^{x} - e^{-x}}{e^{x} + e^{-x}}$ ------------ (S3)	[63]
**Swish**	$y = \frac{x}{1+e^{-x}}$ ------------------------------ (S4)	[31]
**GELU (Gaussian ELU)**	$y = x\Phi(x)$, where $\Phi(x)$ is the Gaussian CDF of x -------------------- (S5)	[32]

Supplementary Table S1: Equations considered for the activation functions.

3. Equations considered for the optimizer function

The optimizer function used in this study is an *adaptive moments estimation* (Adam)-based optimizer [33], which is directly imported from the TensorFlow Keras package. The hyperparameters utilised in this optimizer are as follows:

Hyperparameter	Discriminator	GAN
**Learning Rate**	0.0001	0.0001
**β1**	0.915	0.915
**β2**	0.9987	0.9897
**ε**	0.001	0.001
**amsgrad**	False	True

Supplementary Table S2: Equations considered for the optimizer function.

4. Formulae for the employed image quality metrics

Four image quality metrics are utilized in the current study – the (i) peak signal-noise ratio (PSNR), (ii) signal noise ratio (SNR), (iii) image mean square error (IMSE) & (iv) structural similarity index (SSIM). The formulae underlying each of these metrics are described below:

a. Peak signal-noise ratio (PSNR)

PSNR may be mathematically defined as follows:

$$\text{PSNR} = 10\ lg(\frac{\text{PV}^2}{\text{MSE}})\ \text{----------}\ \text{(S6)}\quad \text{(Source: [64])}$$

where PV is the peak (max) value of a pixel in an image (e.g. 255 for an 8-bit image), MSE is the mean squared error and $lg(x) = \log_{10}(x)$ (for some constant x). Generally, images having a PSNR >20 imply good (noise-free) images, while those with PSNR values between 18-20 indicate an acceptable image quality standard, although the background of the image is likely polluted with noise.

b. Signal-noise ratio (SNR)

Another widely-employed image quality metric (akin to PSNR) is SNR, which may be expressed mathematically as follows:

$$\text{SNR} = 10\ lg(\frac{S}{N})\ \text{-------------------}\ \text{(S7)}$$

where S and N are the respective strengths of the signal and noise [65]. Here, the MATLAB function **psnr(*A*, *ref*)** also returns the SNR value of the noisy image *A* (with respect to *ref*) [64].

c. Image mean square error (IMSE)

IMSE represents yet another quantitative approach for comparing 2 images. IMSE may be computed through the following equation (S8):

$$\text{IMSE}(A, ref) = \sum_{i=1}^{n}(A - ref)^2/n\ \text{------------------}\ \text{(S8)}$$

where an image *A* is being compared against a separate reference (*ref*) image of a similar size (*n* is the total number of pixels in either *A* or *ref*). The MATLAB-implementation of IMSE using the in-built function **immse** [66] is being employed in this context.

d. Structural similarity index (SSIM)

SSIM (the last of the 4 image quality metrics used in the present study) considers 3 factors of imaging, i.e. (i) luminance ℓ, (ii) contrast c and (iii) structure s [67]. In this respect, SSIM has been determined to be generally superior to other metrics (such as MSE) for comparing different images experiencing varying levels of distortion [68]. Mathematically, SSIM may be expressed as follows (adapted from [67]):

$$\mathrm{SSIM}(A, ref) = [\ell(A, ref)]^{\alpha} \bullet [c(A, ref)]^{\beta} \bullet [s(A, ref)]^{\gamma} \text{ --------------------- (S9)}$$

where μ_A, μ_{ref}, σ_A, σ_{ref}, and σ_{Aref} denote the local means, standard deviations, and cross-covariance for images *A, ref* respectively, and

$$\ell(A, ref) = \frac{2\mu_A\mu_{ref} + C_1}{\mu_A^2 + \mu_{ref}^2 + C_1}, \quad c(A, ref) = \frac{2\sigma_A\sigma_{ref} + C_2}{\sigma_A^2 + \sigma_{ref}^2 + C_2}, \quad s(A, ref) = \frac{\sigma_{Aref} + C_3}{\sigma_A\sigma_{ref} + C_3} \text{ ------------ (S10) (Source: [67])}$$

By default, MATLAB assigns $\alpha = \beta = \gamma = 1$ and $C_3 = C_2 / 2$, thereby reducing SSIM to the following:

$$\mathrm{SSIM}(A, ref) = \frac{(2\mu_A\mu_{ref} + C_1)(2\sigma_{Aref} + C_2)}{(\mu_A^2 + \mu_{ref}^2 + C_1)(\sigma_A^2 + \sigma_{ref}^2 + C_2)} \text{ ---------------------- (S11) (Source: [67])}$$

Computation of these four metrics for the images analyzed in the present study were performed in MATLAB R2022B (© 1984-2022, The MathWorks, Inc).

5. ER Figures

The figures shown below are the detailed representations from which Figures 3, 4, 6 & 7 are extricated from.

a. ER-DIC images [training images dataset]

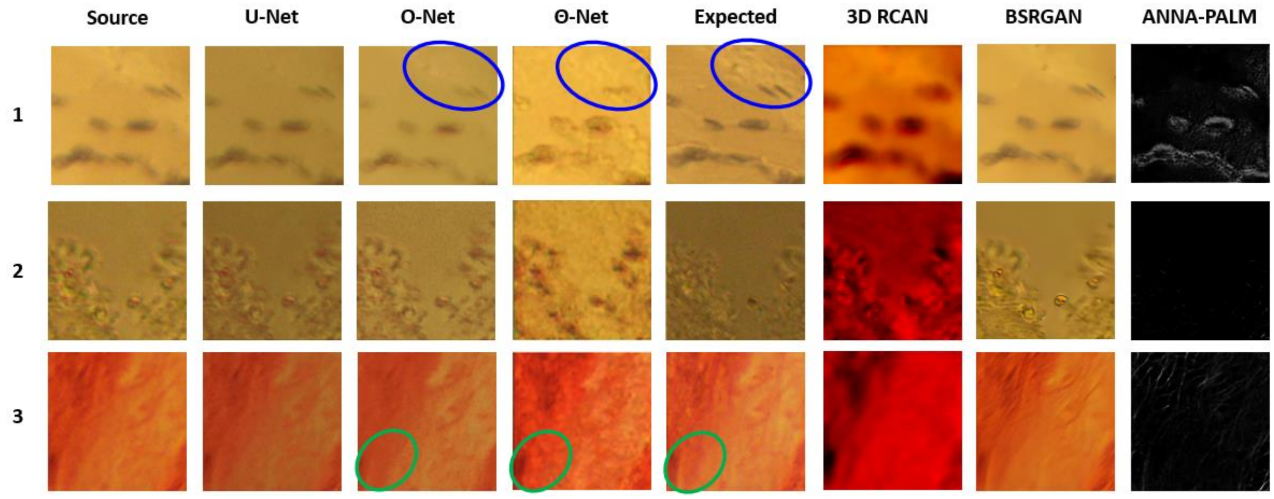

Supplementary Figure S11: Figure illustrating the model-generated images for the DIC training images dataset presented in this study. Row 1 illustrates highly magnified views of skeletal muscle tissue (L.S.), Row 2 shows the periphery of *Amoeba proteus* sp. & Row 3 depicts a sample of cardiac muscle tissue (L.S.). Images generated via models adopting the 3D RCAN, BSRGAN & ANNA-PALM architectures are also included in this Figure for comparison purposes.

b. ER-DIC images [validation (untrained) images dataset]

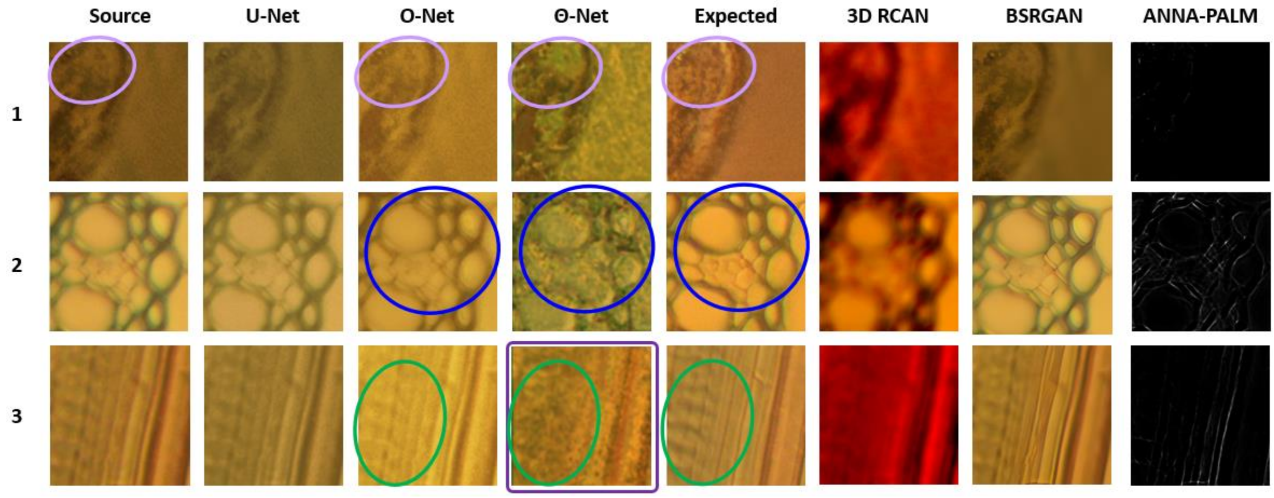

Supplementary Figure S12: Figure illustrating the model-generated images for the DIC validation dataset presented in this study. As with Figure S11 previously, the images generated via models adopting the 3D RCAN, BSRGAN & ANNA-PALM architectures are also included in this

Figure for comparison purposes. The Θ-Net models utilized for generating the ER images in this Figure do *not* employ node scaling (differing from that used for Figure 3), with the exception of the image in the 3rd row (purple-boxed). Brightness enhancement (in MS PowerPoint) was also applied to the first Θ-Net-derived image (Row 1), to make it easier to discern the features.

c. ER-PCM images [training images dataset]

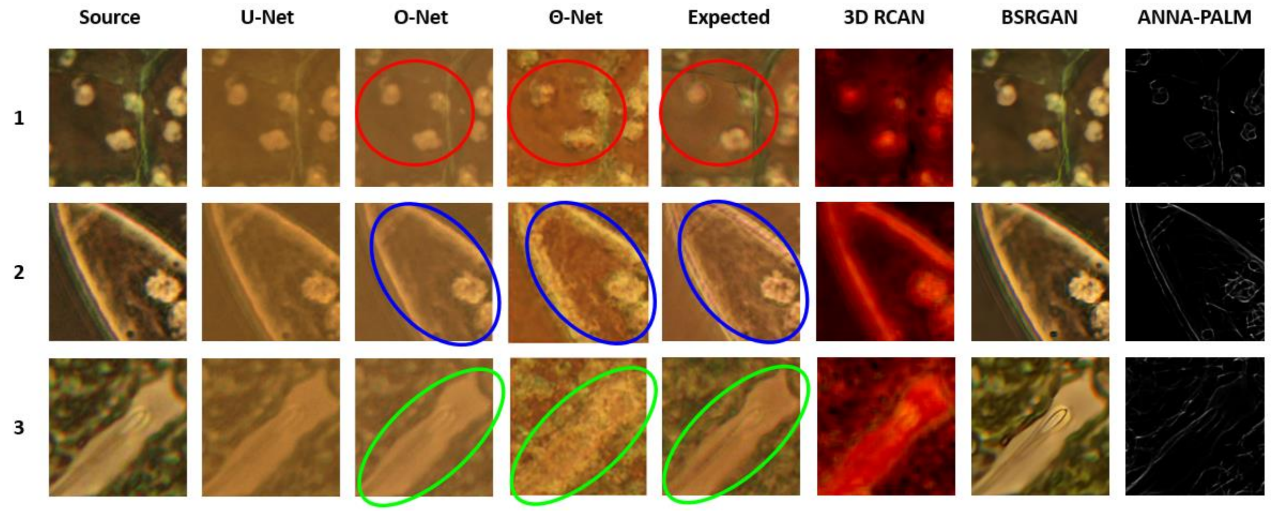

ⓘ **Supplementary Figure S13**: Figure illustrating the model-generated images for the PCM training images dataset presented in this study. 3D RCAN, BSRGAN & ANNA-PALM-based models have their generated images also portrayed alongside those generated via the O-Net & Θ-Net models. Here, it is evident that the red-, blue- and green-encircled regions of the Θ-Net model-generated images depict a greater level of detail (as compared to its O-Net counterpart). Basic contrast enhancement was also applied to *all* of the Θ-Net model-derived images in MS PowerPoint, to make it easier to discern the features.

d. ER-PCM images [validation (untrained) images dataset]

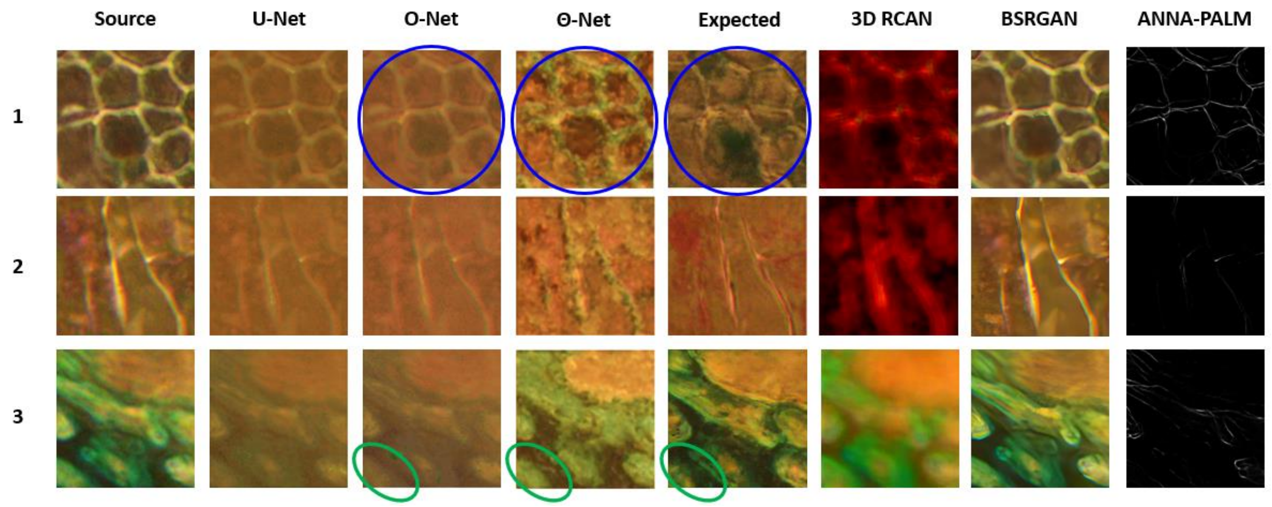

Supplementary Figure S14: Figure illustrating the model-generated images for the PCM validation dataset utilized in this study. Here too, the images generated via models adopting the 3D RCAN, BSRGAN & ANNA-PALM architectures are presented for easy comparison purposes. Basic brightness enhancement (in MS PowerPoint) was applied to *all* of the Θ-Net-derived images, facilitating discernment of the features.

6. Models, Codes & Figures Availability

The models, codes and images utilized in the present study may be downloaded via the link below:

Download link:

https://drive.google.com/file/d/1J0HYPE6-tOJOJ9F_fkifzomQ6oFxJ-Mc/view?usp=sharing

All codes are written in Python or MATLAB – the former having the **.py** extension, while the latter has the **.m** extension. It is recommended that the reader intending to execute the supplied codes for validation use Python ⩾ 3.8 and MATLAB ⩾ R2020a to do so. The model files used for the generating the images depicted in the present study have a **.h5** extension.

7. References

62. “LeakyReLU layer,” Keras, [Online]. Available: https://keras.io/api/layers/activation_layers/leaky_relu/. [Accessed 29 10 2022].
63. “Layer Activation Functions,” Keras, [Online]. Available: https://keras.io/api/layers/activations/. [Accessed 29 10 2022].
64. “Peak signal-to-noise ratio (PSNR),” © 1994-2022 The MathWorks, Inc., [Online]. Available: https://www.mathworks.com/help/images/ref/psnr.html. [Accessed 29 10 2022].
65. C. P. Solutions, “What is Signal to Noise Ratio and How to calculate it?,” © 2022 Cadence Design Systems, Inc., [Online]. Available: https://resources.pcb.cadence.com/blog/2020-what-is-signal-to-noise-ratio-and-how-to-calculate-it. [Accessed 29 10 2022].
66. “Mean-squared error,” © 1994-2022 The MathWorks, Inc., [Online]. Available: https://www.mathworks.com/help/images/ref/immse.html. [Accessed 29 10 2022].
67. “Structural similarity (SSIM) index for measuring image quality,” © 1994-2022 The MathWorks, Inc., [Online]. Available: https://www.mathworks.com/help/images/ref/ssim.html. [Accessed 29 10 2022].
68. Z. Wang, A. C. Bovik, H. R. Sheikh and E. P. Simoncelli, “Image Quality Assessment: From Error Visibility to Structural Similarity,” IEEE Transactions on Image Processing, vol. 13, no. 4, pp. 600-612, 2004.
